# Desire to Quit Smoking, Opt-Out Tobacco Treatment, and Cessation: A Secondary Analysis of a Randomized Clinical Trial

**DOI:** 10.1001/jamanetworkopen.2024.33802

**Published:** 2024-09-17

**Authors:** Byron Gajewski, Babalola Faseru, Kimber P. Richter

**Affiliations:** 1 Department of Biostatistics and Data Science, University of Kansas School of Medicine, Kansas City; 2 Department of Population Health, University of Kansas School of Medicine, Kansas City; 3 University of Kansas Cancer Center, Kansas City

## Abstract

This secondary analysis of a randomized clinical trial examines whether an individual’s desire to quit interacts with the association between opt-out tobacco treatment and 1-month quit rates.

## Introduction

A randomized clinical trial (RCT) demonstrated that opt-out treatment achieved higher verified quit rates (22%) than opt-in treatment (16%) 1 month after randomization.^1^ When people who smoke have a higher desire to quit before treatment, they have a higher rate of smoking cessation.^2^ Understanding how individuals respond to different smoking cessation treatment approaches could guide clinical personalized decision-making. We wanted to know if the opt-out effect was diminished if the participant had less desire to quit when initially approached.

## Methods

The parent study for this secondary analysis of an RCT (not prespecified) was a bayesian adaptive population-based RCT among people who smoked who were patients at a hospital. The trial protocol was previously published (Supplement 1).^1^ Patients were randomized to opt-out treatment (counselors and medical staff provided inpatient nicotine replacement therapy, prescriptions for postdischarge medications, a 2-week medication starter kit, treatment planning, and 4 outpatient counseling calls) or opt-in treatment (counselors and medical staff provided the same only to patients willing to quit) and consented to participation at a 1-month follow-up. For patients receiving opt-out treatment, staff arranged for inpatient medications to manage withdrawal, completed a treatment plan with patients, assisted patients in selecting a cessation medication for prescription on discharge, and described outpatient follow-up. Unless patients opted out of all elements of the treatment plan, they received all elements of care. Patients in the opt-in group were offered and received the elements of care they opted in to receive. The original study was approved by the University of Kansas Medical Center Institutional Review Board and followed the CONSORT reporting guideline.^1^ The University of Kansas Medical Center determined that this study did not require additional review, approval, or consent given that data were deidentified.

**Figure 1.**
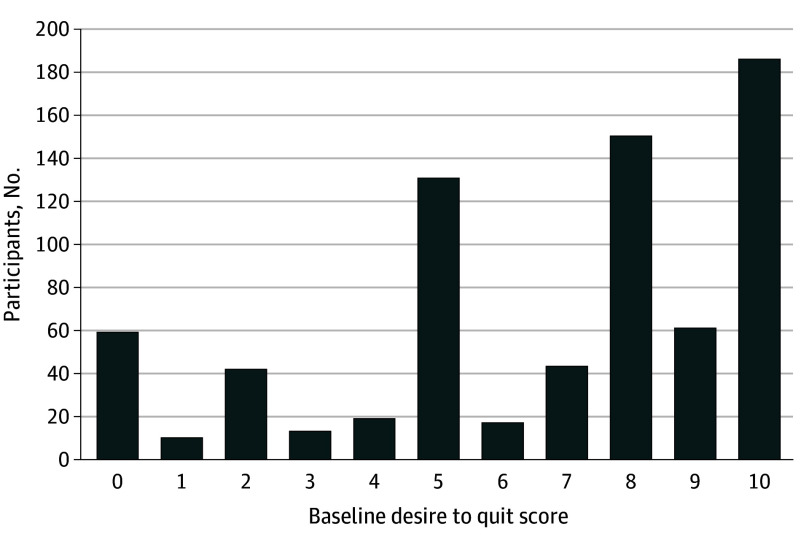
Baseline Desire to Quit Smoking

Patients were asked to “Choose a number between zero and ten that indicates where you are now at in thinking about quitting smoking, 0 = I have no thoughts about quitting smoking, 10 = I am taking action to quit smoking” (Figure 1). We assessed the association of the desire to quit and its interaction with the group with month 1 of abstinence using bayesian logistic regression. We explored multiple models with various complexities, including a normal dynamic linear model (NDLM), an aggregate model, an NDLM but with a fixed opt-out effect, a separate model, and a pooled model. We selected the best model (ie, with the lowest deviance information criteria) for final inference. Analyses were conducted for consented participants only using OpenBUGS software version 3.2.3 revision 1012 (OpenBUGS Foundation). For each level of desire to quit smoking, we calculated the 1-sided posterior probability that opt-out was better than opt-in treatment. The level of significance was 5% in the original trial design.

## Results

**Figure 2.**
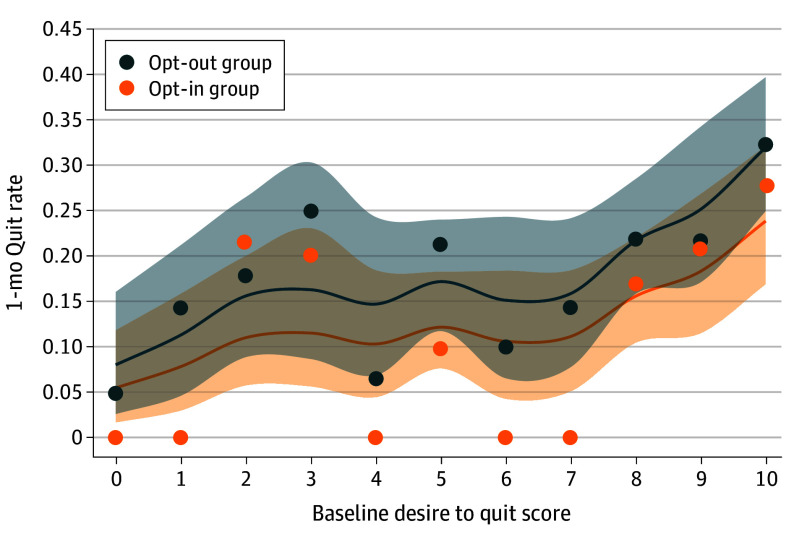
Normal Dynamic Linear Model of Opt-Out Effect Opt-in and opt-out groups are shown. Circles indicate observed rates.

Among 739 participants (mean [SD] age, 51.4 [14.7] years; 349 females [47.2%]), as the desire to quit increased, smoking abstinence rates increased. However, the treatment effect of opt-out treatment stayed at a posterior probability of 0.976 (opt-out at level 0 desire to quit, 8% [95% CI, 3%-16%]; opt-out at level 10 desire to quit, 32% [95% CI, 25%-40%]). Figure 2 shows the NDLM with a fixed-effect opt-out effect for both groups.

## Discussion

In this secondary analysis of an RCT, the lower baseline desire to quit smoking had an approximately 25–percentage point absolute lower abstinence rate than the highest desire to quit smoking. The opt-out effect was not diminished if the participant had less desire to quit when initially approached. These findings may inform clinical decision-making. Even though people with a lower desire to quit had lower quit rates, the advantage of opt-out treatment was constant. This suggests that people may benefit from opt-out treatment regardless of their level of desire to quit. This study was limited to people who were hospitalized and smoked from a Midwestern hospital, and the outcome was relatively short, at 1 month.

## Notes

Supplement 1.
Trial Protocol and Statistical Analysis Plan

Supplement 2.
Data Sharing Statement
