# Supplementary material for: Desire to Quit Smoking, Opt-Out Tobacco Treatment, and Cessation: A Secondary Analysis of a Randomized Clinical Trial
Source: JAMA Netw Open. 2024 Sep 17;7(9):e2433802. doi: 10.1001/jamanetworkopen.2024.33802 (PMC11409148; doi:10.1001/jamanetworkopen.2024.33802)
Supplement: Supplement 1. — Trial Protocol and Statistical Analysis Plan [file jamanetwopen-e2433802-s001.pdf]

Supplement 1
Full Study Protocol
CHANGING THE DEFAULT FOR TOBACCO TREATMENT

HYPOTHESIS AND SPECIFIC AIMS

Globally, one billion people will die from tobacco-related illnesses this century. Most health care providers, however, fail to initiate medication therapy or cessation support with their smokers, even where these resources are readily available. This may in part be due to the cessation treatment "default." Current treatment guidelines recommend that providers a) ask patients if they are willing to quit and b) provide cessation-focused medications and counseling *only* to smokers who state they are willing to quit.

For other health conditions—diabetes, hypertension, asthma, and even substance abuse—the treatment default is to a) identify the health condition and b) initiate evidence-based treatment. For example, when patients are newly diagnosed with diabetes, they aren't asked if they are 'willing' to start treatment. The physician simply begins with a discussion of treatment options. As with any healthcare treatment option, patients are free to decline—they can "opt out" if they wish to refuse care. If patients do nothing, they *will* receive care. For tobacco users, however, the default is that they have to "opt in" to receive cessation assistance: providers ask smokers if they are willing to quit, and only offer medications and cessation support to those who say "yes". This drastically limits the reach of cessation services because, at any given encounter, only 1 in 3 smokers say they are ready to quit. As a result, few receive medications or cessation counseling. Recent studies suggest that, when provided with cessation medications and counseling, "unmotivated" smokers are as likely to quit as "motivated" smokers. Hence, there is a *critical need* to examine the impact of changing the treatment default on utilization and quitting.

Our long-term goal is to save lives through novel systems of evidence-based care delivery. The objective of this application is to determine the impact of providing all smokers with cessation pharmacotherapy and counseling unless they refuse it (**OPT OUT**) versus current practice—screening for readiness and only offering cessation assistance to smokers who say they are ready to quit (**OPT IN**).

Our central hypothesis is that **OPT OUT** will result in significantly higher rates of medication use, receipt of cessation counseling, and cessation compared to **OPT IN**. Tobacco treatment is an excellent policy arena for testing the effects of changing the treatment default because most smokers report they would ultimately like to quit smoking. Paradigm-shifting research on organ donation, and numerous other studies, demonstrate that changing defaults dramatically changes decisions, behaviors, and outcomes. The default is created by the way choices are presented. Changing the way providers offer treatment could harness the ambivalence smokers feel about smoking and "nudge" them toward accepting treatment and quitting.

We will conduct the trial in an academic hospital. The study is a posttest-only randomized clinical trial with delayed informed consent. For **OPT IN**, clinicians will, in accordance with current guidelines, ask smokers if they are ready to quit. With smokers who say "yes", clinicians will ask if they would like cessation medication and/or referral to the state tobacco quitline. Smokers will receive each component of care to which they opt in. With smokers who say "no" clinicians will provide brief motivational counseling designed to increase readiness to quit. For **OPT OUT**, clinicians will explain that the hospital provides free tobacco treatment to all smokers, provide the smoker with cessation medications, and refer all smokers to the quitline. As with all medical care, smokers in **OPT OUT** will be free to decline any aspect of treatment.

The trial employs a Bayesian adaptive design study—an efficient and ethical strategy for comparative effectiveness clinical trials design, because it allocates more patients to effective treatments. We project that 1,000 patients, 500 in each arm, will be required to test differences at 1- and 6-months post randomization. The study has the following aims and hypotheses:

First Aim: To determine the population impact of changing the default for tobacco cessation treatment.
<u>Hypothesis 1</u>: Significantly more patients in **OPT OUT** will participate in counseling, use cessation medications, and be abstinent from smoking at 1 month post-randomization compared to **OPT IN**.
Second Aim: To identify long-term impact, mediators of effectiveness, and costs.
<u>Hypothesis 2</u>: Significantly more smokers in **OPT OUT** will be abstinent from smoking, and mediation

analyses will find that counseling participation, medication use, and default-theory based variables will partially or fully explain the effect of **OPT OUT** on cessation at 6 months post-randomization.

Hypothesis 3: **OPT OUT** will be more costly—in terms of upfront costs—but will be more effective than **OPT IN.** As a result, **OPT OUT** will be more cost-effective from a provider perspective.

This is a population-based study that targets an endpoint of vital interest; applies minimal eligibility criteria to broaden generalizability; and utilizes hospital staff for interventions to ensure long-term sustainability. The study employs a highly innovative design to evaluate a major shift in our approach to care. If effective, this change would expand the reach of tobacco treatment from 30% to 100% of smokers.

SIGNIFICANCE

Based on current rates of tobacco use uptake and cessation, twenty million Americans will die from tobacco related illnesses between 2000-2050.[1] Due to the 20-40 year time lag between starting smoking and the onset of tobacco related illnesses[2], helping smokers quit is the best way to immediately reduce illness and deaths.

Guidelines-based tobacco treatment consists of two branches[3]: motivational counseling, based on principles of Motivational Interviewing[4], for patients not willing to make a quit attempt; and cessation-oriented pharmacotherapy and counseling for smokers who are prepared to make a quit attempt. The goal of motivational treatment is focused on building smokers' motivation to quit; findings on its effectiveness for smoking cessation are, at best, mixed.[5]

Cessation-oriented pharmacotherapy and counseling doubles or even triples quit rates over no-treatment controls,[3,6] but the 'default option' for smoking cessation is 'no treatment'. With each step of guidelines-based tobacco treatment, smokers must say 'yes' (*they would like to quit, would like to use medications, would like counseling*) in order to receive evidence based care—they must 'opt in'. This is in stark contrast to treatment of other common medical conditions, such as hypertension, where the default option is 'treatment': physicians identify and initiate treatment for high blood pressure[7], and patients must 'opt out' if they don't want care.

Consequently, less than 1 in 5 smokers actually get assistance in quitting on any given outpatient encounter with their health care providers.[8-10] In hospitals, an even smaller percentage of smokers receive assistance: a meta-analysis found that only 14% of inpatient smokers were provided with a prescription for cessation medication and 12% received referrals for follow up.[11] Moreover, because smokers must opt in for cessation counseling and medication separately, few receive both components of evidence-based care.[12] Many would blame this treatment gap on smokers and their lack of motivation to quit smoking. This application, and a peer-reviewed article prepared in tandem with this application[13], proposes that smokers fail to receive effective cessation treatment due to how the U.S. structures the tobacco treatment default. The application examines the effects of pro-actively providing cessation-oriented treatment to all smokers.

How Do Defaults Influence Health Choices?

For any given choice, there is a default option—the option that will occur if the chooser does nothing.[14] For most chronic health conditions—diabetes, hypertension, asthma, and even substance abuse—guidelines direct health care providers to identify the health condition and initiate treatment.[7,15,16] As with any healthcare treatment option, patients are free to refuse.

In this manner, healthcare providers act as "choice architects" by creating a context in which the patient is presented with, and makes, a decision.[14] Defaults are unavoidable in any context because there must be a rule that determines what should occur at decision points should no action be taken—consider for example computer configuration defaults and defaults on annual health insurance options. In healthcare, most treatment guidelines direct clinicians to provide evidence-based treatment, which the patient will receive by default unless she or he refuses treatment. In fact, this choice architecture is arguably the most ethical. Where there is strong clinical evidence that support an appropriate therapy, that therapy should be presented as the default.[17] Hence, the exceptional position that smokers should be asked if they are "ready" to quit creates a rate-limiting step in tobacco treatment that should be critically examined.

A wide range of studies demonstrate that defaults powerfully affect choices and behaviors. Changing default options changes consumers' choices of health care plans[18], information shared on the internet[19], and organ

donation[20]. Opt out policies for students and parents greatly facilitate participation in health and educational interventions and research.[21] Disney World changed its default sides for kids meals to include apples and milk rather than fries and soft drink; although pre-change data are not available, 65% of children now receive apples and 68% milk following the change.[22]

Although choice research is a relatively new field, defaults are hypothesized to be highly influential because they capitalize on *implied recommendations for courses of action* and *status quo biases*. For example, the way a healthcare provider presents a choice may "leak" information about the provider's attitudes toward options as well as their implied recommendation for a course of action.[23] Provider preferences could be leaked by tone of voice, phrasing of options, order of options, omission of options, or what option is presented as the default option. In addition, people making decisions consistently exhibit a status quo bias—when an opportunity exists to either do something or do nothing, people tend to do nothing.[18] Taken together, information leakage by choice architects and status quo biases on the part of decision makers can tip the balance in favor of some choices, and against others. Regardless of how carefully or thoughtlessly options are presented, they can have a powerful impact on choices.[14]

Decision theorists suggest that institutions should structure default choices to be the options that make the choosers better off, as judged by themselves.[24] Tobacco treatment is an excellent candidate for a default that favors treatment because 70% of smokers state they ultimately want to quit smoking.[12] Choosing to quit in the near future, however, is extremely difficult for smokers because they get the pleasures of smoking, and the pain of abstaining from smoking, in the present but suffer the terrible consequences of smoking in the future.[14] This may be why fewer smokers state that they are ready to quit in the near future. They could use a "nudge" to accept treatment in order to reach their ultimate goal of quitting.

Research Contribution

The contribution of this trial will be to identify the impact of default tobacco treatment, for an entire population of smokers, on treatment uptake and cessation. It will identify the costs and cost-effectiveness of changing the default, and examine psychological mediators of change based on decision theory. This contribution is significant because it will definitively determine the impact of routinely assessing smokers' readiness to quit in real world clinical practice. Assessing readiness is an integral step in the "5 As" of tobacco treatment *(Ask, Advise, Assess [readiness to quit], Assist, Arrange)*.[3] Currently, the *Assess* step is supported by "C" level evidence of effectiveness, as there are no trials that establish that it has a positive impact on cessation.[3] Because most (70%) of the 46 million adult U.S. smokers will visit a health care provider in a given year,[25,26] study findings will either support—or dramatically change—a clinical practice that affects treatment and outcomes for millions of patients per year.

The benefits that flow from this contribution are significant. Regardless of outcome, the trial will provide a model of how to alter and evaluate the impact of health care defaults. If OPT OUT proves to be more effective, it will expand the population eligible for cessation treatment by over 300%--from 30% to 100% of smokers. It will also simplify the tobacco treatment algorithm, and relieve busy health care providers of the burden of evaluating patients' readiness to quit.

Evidence for Study Components, Theoretical Model, and Design Considerations

This study tests the utility of assessing readiness for tobacco treatment. In conducting the trial we adhere to evidence-based recommendations for treating hospitalized smokers. We will provide post-discharge counseling. We will provide nicotine replacement for post-discharge medication. Our intervention will operationalize key features of treatment defaults. To assess mediating effects, we will measure patient perceptions of these features. We review each of these areas below.

Guidelines-based tobacco treatment. Clinical Practice Guidelines for Treating Tobacco Use and Dependence[3] recommend providers use the "*5 As*" to intervene systematically with patients: *Ask* the patient if she or he uses tobacco; *Advise* him or her to quit; *Assess* willingness to quit; *Assist* with quit attempt using patient-centered counseling and pharmacotherapy; and *Arrange* for follow-up to prevent/address relapse. Guidelines-based counseling strategies for smokers *ready* to quit include developing a quit plan, discussing and selecting medications, and providing practical counseling that includes problem solving and skills training; effective support

may be provided by providers or tobacco quitlines. The highest abstinence rates are achieved with a combination of counseling and pharmacotherapy.[27]

Recommended counseling for smokers *not* ready to quit employs a Motivational Interviewing approach and covers the “5 Rs”: *Relevance*—address why quitting is personally relevant; *Risks*—explore the risks of continuing to smoke; *Rewards*—explore the potential rewards of quitting; *Roadblocks*—explore barriers to quitting; and *Repetition*—repeat motivation each time the patient visits the healthcare setting.

The intervention includes one study arm (OPT IN) that follows all 5 As, and one study arm (OPT OUT) that omits the *Assess* step. In the OPT IN arm, patients will be screened for readiness. Those who state they are ready to quit (i.e., who “opt in” to treatment) will be referred to outpatient counseling and sent home with a 14-day ‘starter pack’ of nicotine replacement (NRT) to bridge their transition to outpatient care. In OPT OUT, all patients will be referred to outpatient counseling and given an NRT starter pack (i.e., patients will have to “opt out” to not receive care). During times when face to face contact with hospitalized patients is not feasible, these starter packs will be mailed to patients’ homes following hospital discharge.

Treatment for both study groups will be consistent with the 2012 Cochrane Review of Interventions for Smoking Cessation in Hospitalized Patients[28] which found that, to be effective, behavioral interventions must start in the hospital and continue at least one month after discharge to be effective. The review also examined the impact of starting cessation medications in the hospital. Among all first line medications (varenicline, bupropion, and NRT), only NRT significantly increased quit rates over counseling alone, which guided our decision to provide NRT to patients in this trial.

Proactive tobacco quitlines. Quitlines are effective and cost effective for smoking cessation.[29,30] They are available, free, to most U.S. smokers[31]; services are delivered via telephone which minimizes many access barriers; hospitals do not incur costs for the services; and many quitlines are undersubscribed and eager to increase their reach.[32,33] We will provides outpatient counseling services that mimic current quitline services. We will provide the outpatient telephone base counseling ourselves since the quitline’s current protocol involves only counseling smokers who state they are ready to quit smoking.

Theoretical model. We hypothesize that smoker perceptions of what course of action their cessation counselor recommends, and what type of treatment is the status quo at the hospital, will affect their counseling participation, medication use, and quit rates (Figure 1). Mediation analyses are described under *Data Analysis*.

Figure 1. Theoretical Model

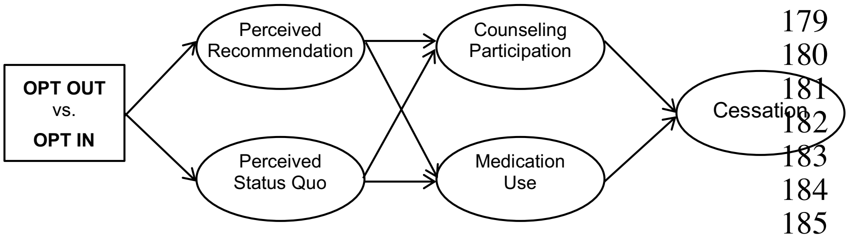

Design considerations: readiness to quit. One possible objection to the proposed study is the widely held belief that smokers must be motivated in order to quit. However, numerous clinical trials have found that smokers who report they are *not* ready to quit actually quit at the same rates as those who report they *are* ready to quit[34,35], possibly because intentions to quit can change rapidly.[36] In fact, a majority of smokers quit as a result of unplanned, spontaneous, quit attempts[37]—which suggests that motivation is highly variable and that other factors may help to trigger quit attempts. Moreover, smokers who are not planning to quit will accept treatment. The population-based Inter99 intervention trial in Copenhagen offered all smokers attending a lifestyle modification consultation the opportunity of enrolling in smoking cessation groups. Even though only 11% had reported they were planning to quit in the next month, over 1 in 4 (27%) enrolled in cessation groups.[38] Thirty-five percent of all participants were abstinent by the end of the groups. Among successful quitters, only 16% were those who had been planning to quit smoking at the beginning of the trial.[34] If Inter99 had followed guidelines endorsed by the Danish Medical Society, it would have restricted its offer of treatment to those few smokers who were planning to quit, and failed to help many achieve abstinence.

A meta-analysis by Aveyard and colleagues suggests that physicians will more effectively treat tobacco dependence by providing opt-out care.[39] Four studies identified in the meta-analysis offered nicotine replacement therapy (NRT) to all participants regardless of readiness to quit. Compared to brief advice alone, smokers who

were also given NRT were 49% more likely to quit (RR 1.49, 95% CI: 1.17-1.89). Another trial examined the effects of opt-out behavioral support for cessation, compared to brief advice to quit.[40] Although opt-out care did not conclusively outperform advice to quit in promoting abstinence (RR 3.10, 95% CI: 0.38-25.51), it did prompt more quit attempts (1.69, 95% CI: 1.24-2.31). Importantly, patients found the provision of behavioral support more helpful than simply being advised to quit.

Design considerations: motivating smokers. Another concern is that many believe that, for unmotivated smokers, tobacco cessation intervention should focus on increasing readiness to quit. However, it is important to first test the assumption that smokers must state they are ready to quit in order to be able to quit. Several non-randomized trials, in secondary analyses, have found that non-motivated participants quit at the same rates as motivated participants.[41] If the present study hypothesis proves correct, simply providing treatment to all smokers will result in higher quit rates. If so, screening for readiness should not be universally performed, as screening for readiness would simply reduce the number of patients receiving treatment. If, however, our hypothesis proves wrong and screening for readiness results in similar or better quit rates, then the next logical and crucial research step indeed will be to develop effective methods for motivating smokers to quit. Either way, the present study addresses the gap in knowledge that must be filled before advancing other strategies to expand the reach of cessation treatment.

Design considerations: providing tobacco treatment, without first asking if the smoker is ready to quit, might be paternalistic or coercive. We believe this is an open question in light of the findings from Slama and colleagues' opt-out treatment for behavioral counseling[40]—that patients found the offer of behavioral support more helpful than simply being advised to quit. Viewed from another perspective, asking patients if they are ready to quit, and only offering medications or cessation-oriented counseling to those who say "yes", could also be considered paternalistic. In order to collect data to address this issue, we will, at 6-month follow-up, ask participants to rate how paternalistic they perceived their hospital tobacco treatment providers to be and will include this in mediation analyses to understand any differences in perceived paternalism between groups and the impact of these perceptions on quit behaviors.

Figure 2. Study flow diagram

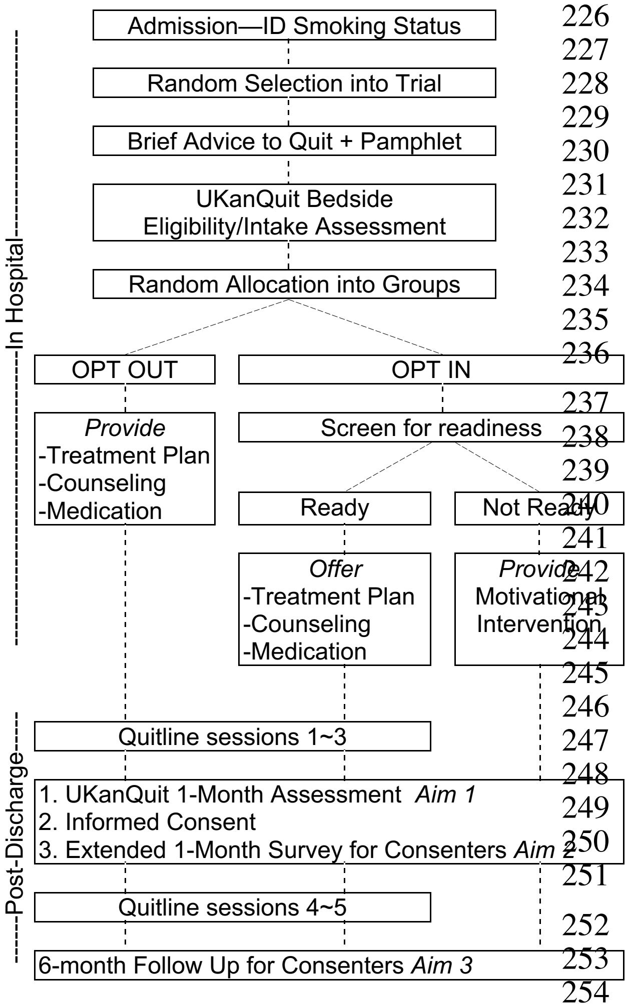

INNOVATION

It is not possible to randomly assign participants to be "ready" or not. Perhaps because motivation is often viewed as an innate characteristic of an individual[42], no experimental studies have examined whether the requirement that smokers be "ready to quit" is a useful step in tobacco treatment. However, recent research on motivation and decision theory suggest that environmental influences such as treatment defaults, the context in which a decision is made, and treatment provider counseling style, have a great deal of influence on individual choices and outcomes.[14,17,20,43]

As described under *Significance*, providers miss many opportunities for treating tobacco dependence, which has created a large treatment gap. This study innovatively reframes this gap as a direct result of our treatment default. Current guidelines recommend providers screen for readiness, which in turn requires smokers to 'opt in' for treatment. Viewed from this perspective, the effects of screening for readiness on tobacco treatment can be tested by changing the way treatment is offered to smokers. The study is also innovative because it identifies a major rate-limiting step in access to tobacco treatment. Regardless of what population, setting, or approach is used to disseminate tobacco treatment, as long as providers screen for readiness, the majority of smokers will report they are *not* ready to quit—and they will *not* receive care.

Other unique features of the trial include the posttest only delayed-consent randomized control group design[44], and Bayesian adaptive trial design.[45] The posttest-only design controls for all of the threats to internal validity that a pretest-posttest design controls for. In addition, it avoids testing effects by minimizing repeated measurement—especially prior to the intervention. This will enable us to see the true effects of changing the default on initial treatment choices. The Bayesian adaptive design study determines our sample size and how patients are allocated to groups.[45] Bayesian designs are becoming widely used in PCORI pragmatic clinical trials, because they are efficient and ethical—they can get answers to research questions early, and they allocate more patients to the better-performing treatment arm (described in detail under *Approach*).[46] The study will also evaluate the impact of psychological mediators on treatment participation and smoking cessation, in order to gain insight into why and how changing the default did—or did not—work.

Features of this application will benefit research far beyond the field of tobacco control. It will provide a model for how to experimentally test the effects of health care defaults. The post-test only design is also extremely useful in examining brief interventions, such as vaccination policies, HIV testing, and procedures that might be included on hospital order sets.[43] Last but not least, patients will benefit by receiving care in institutions that critically examine how they present choices to patients, and ensure those "choice architectures" are designed to help patients reach their long-term and short-term health goals.

RESEARCH STRATEGY

Overview

In the 6-month planning phase, we will finalize all protocols, data collection forms, and ethics approvals. We also will train participating hospital staff. The trial employs a 2-arm design with individual randomization to groups (Figure 2). After patients are admitted to the hospital, research staff will identify every smoker via the electronic

health record (EHR), randomly select the daily quota of study participants, and provide the quota to UKanQuit hospital staff. At the bedside, or over the telephone during the COVID-19 pandemic when staff are unable to meet at the bedside, UKanQuit staff will briefly advise all smokers to quit, provide an 8-page pamphlet on cessation resources, and use a tablet computer to randomly assign patients to one of 2 study arms: OPT OUT or OPT IN. UKanQuit staff will administer the appropriate intervention. UKanQuit staff will collect contact information for their 1-month post treatment phone assessment, which is standard practice for the service and accords with Joint Commission follow-up recommendations.

After discharge, UKanQuit staff will contact all smokers seen. They will collect month 1 service data, describe the research study, collect verbal consent and transfer patients to research staff extended month 1 data collection. Research staff will conduct a similar assessment at month 6 among consenting patients. Outcomes include biochemically-verified 7-day point prevalence abstinence at 1 and 6 months, post-discharge counseling utilization, and medication utilization. We will also assess psychological mediators and cost-effectiveness. We estimate 1,000 participants, 500 in each arm, will be required to detect the expected treatment effect on abstinence.

Preliminary Studies

The study is highly feasible due to the unique relationship the research team has with the University of Kansas Hospital and the expertise our team has in conducting hospital-based research.

UKanQuit clinical service. The University of Kansas Hospital founded UKanQuit in 2006, and operates it via a contract with the Department of Preventive Medicine and Public Health (Service Directors Richter and Ellerbeck). UKanQuit is fully integrated into hospital protocols and electronic systems. In its first 6 years of operation it treated 7,700 smokers. UKanQuit has been the subject of two quality improvement studies[47,48] and has served as the platform for two clinical trials (EQUIP and a PCORI trial, P.I. Ellerbeck). UKanQuit demonstrates our ability to work in hospitals and collect data from clinical services.

Enhancing Quitline Use among Inpatients (EQUIP). (U01 HL105232, Richter, P.I.). EQUIP built on the UKanQuit clinical service to test two novel ways of linking smokers with post-discharge care: 1) fax-referring smokers post-discharge to the state quitline versus 2) providing an inpatient “warm handoff”—linking smokers to the quitline while they are still in the hospital by calling the quitline at the patients’ bedside and having the patient enroll and complete their first counseling session during their hospital stay[49]. We hypothesize the warm handoff will enroll twice as many smokers in quitline services and result in much higher quit rates than fax referral. EQUIP employs many of the procedures that we will use for the present study, including utilization of UKanQuit staff for identifying and intervening with smokers, bedside randomization via computer tablet, referral to quitline, collaboration with quitline vendors and the state purchasers of quitline services, phone-based research follow up at 1 and 6 months, verification of cessation via mailed salivary cotinine and/or proxy, and assessment of costs for cost-effectiveness analyses. EQUIP recruited 1,054 Kansas smokers; month 1 follow up rates were 89%; month 6, 85%. EQUIP demonstrated the team has the expertise and tools to successfully conduct the present project.

Proactive versus Reactive Referral to Quitline.[50] This pilot project tested the effects of OPT IN (reactive) versus OPT OUT (proactive) for referral to counseling via the state tobacco quitline. We employed a pre-test, post-test comparison group design in which a script-based OPT IN approach was employed over a two-week period and a script-based OPT OUT approach was employed in the following two-week period. Scripts were inserted into the counseling protocols of the UKanQuit inpatient treatment program. The OPT IN group had an overall quitline enrollment rate of 36% (9/25). The OPT OUT group achieved an overall enrollment rate of 55% (11/20). This pilot study suggests that smokers at all levels of readiness are willing to enroll in treatment, and that providing OPT OUT quitline referral expands the reach of quitline counseling.

Motivational Interviewing and Smoking Cessation.[51] Team investigators Richter, Catley, and Ellerbeck conducted a clinical trial that compared motivational interviewing (MI) to brief advice (BA) and health education (HE) for motivating quit attempts among smokers who were not ready to quit (R01 CA133068; P.I. Catley). Participants had very low (1.9 on a 0-10 scale) level of readiness and motivation to quit. Participants in BA received one brief in-person session while those in MI and HE received 4 individual time-matched counseling sessions over 18 weeks. Surprisingly, even in this low-motivated sample, the proportion of quit attempts at 24 weeks post randomization ranged from 45% (BA) to 61% (HE) and did not differ significantly across the three study groups.

Rates of cessation were also equivalent across groups. This study suggests that even brief advice is powerful enough to prompt high rates of quit attempts among unmotivated smokers, which supports the hypothesis that an opt-out treatment default will lead to high rates of treatment uptake and cessation.

Other collaborations. Ellerbeck (P.I.) and Richter (Co-I) are collaborating on two NIH-funded studies (R01CA101963, Ellerbeck P.I.; R01-HL08764301; Richter, P.I.) that are set respectively in Kansas hospitals and Kansas primary care clinics. Dr. Shireman is co-investigator on both of our hospital-based studies, managing their respective cost-effectiveness analyses. This demonstrates our strong collaborative history and ability to conduct research with quitline services in a broad range of clinical care settings.

Study Planning

Current practice, UKanQuit hospital tobacco treatment service. UKanQuit hospital staff currently treat all hospitalized smokers that request treatment, or whose physicians have ordered treatment, via the EHR. UKanQuit policy is to first address smokers' comfort before raising the subject of quitting. At the hospital bedside, or during the COVID-19 pandemic when staff were unable to meet at the bedside then over the telephone, UKanQuit staff greet patients, confirm they are smokers, and assess/address current level of craving and withdrawal. Staff immediately work with patients' nurses and physicians to order/increase nicotine replacement or other medications to reduce abstinence-induced discomfort. UKanQuit has found this disarms any potential resistance from patients and leads to very low refusal rates. For example, in the most recent quarter of operations, staff approached 349 patients but only 5 (1%) refused care (internal data). UKanQuit staff then collects service intake data, provides brief, personalized advice to quit as recommended by current treatment guidelines[3], and provides the UKanQuit service pamphlet that describes the risks of smoking, the benefits of quitting, the effectiveness of counseling and medications, and resources for quitting (*Appendix 1*).

Current UKanQuit tobacco treatment adheres to current guidelines and corresponds to the OPT IN (control) condition of this trial. Staff screens for readiness to quit, provides motivational counseling to smokers who are *not* ready to quit, and provides assistance in quitting to smokers that *are* ready to quit. Motivational intervention involves engaging patients in a discussion of the guidelines-recommended "5 Rs" (*Relevance, Risks, Rewards, Roadblocks, Repetition*) (see *Motivational Counseling Checklist, Appendix 2*).[3] For patients ready to quit, treatment consists of brief practical counseling[3], including problem solving barriers to quitting, identifying sources of support and discussing how quitline counseling works, and discussing and selecting medications. Staff complete a treatment plan with patients (*Appendix 3*) that documents reasons for quitting, the patients choice of post-discharge medication, and sources of support, including referral to the state tobacco quitline. All treatment is recorded in the hospital EHR using "smart notes"—drop-down menus using controlled vocabulary to ensure consistent documentation. Recommendations for discharge medications are provided to the patient care team, and UKanQuit staff fax-refer to the quitline all patients who accept quitline services. At one month post-discharge, in accordance with Joint Commission guidelines, UKanQuit staff call all smokers to assess tobacco use status, quit attempts, and treatment adherence. Under *Clinical Trial Phase (below)*, we describe how we will augment UKanQuit protocols to test the OPT OUT approach.

UKanQuit staff training. UKanQuit staff have masters' degrees in healthcare or addictions case management and counseling. All have completed intensive tobacco treatment training and one counselor is fluent in Spanish. UKanQuit staff are familiar with quitline fax-referral procedures as this is one of the study arms in EQUIP. Training will include lecture, demonstration, and practice between staff, mock patients, and actual hospital patients. Dr. Cately from Children's Mercy Hospitals and Clinics has a PhD in Counseling Psychology and will lead the development of the final treatment protocols and will assist in the development of tools to assess counselors' fidelity in delivering the OPT IN or OPT OUT treatment protocols. Staff will provide input into the final treatment protocols, which will be codified into checklists that will be included in treatment packets and used for fidelity monitoring. The training will be incorporated into UKanQuit staff weekly team meetings, will span 4-6 weeks of 30-minute sessions per week, and will include homework on practicing verbal consent, OPT OUT, and OPT IN procedures. Staff will be considered trained to criteria when they are able to correctly perform all components of both conditions with three exemplar patients. Fidelity monitoring will be used to provide ongoing feedback and a summative assessment at the end of the trial.

Study Design Considerations

Overall study design and setting. We considered conducting a pre-test, post-test control group design. This common design collects data from participants at the outset of the trial and would require patients to consent to study procedures before enrolling in the trial. This would, however, require patients to "opt in" to treatment intervention. Pre-trial assessment and consent might discourage some smokers from participating. We rejected this design, as it is the very experimental condition we seek to test. Our IRB and the hospital are supportive of this approach as it is will not adversely impact participants and is consistent with current UKanQuit practice and U.S. treatment guidelines (see *Human Subjects* and *Letters of Support*).

Measuring main outcome at 1 month post-intervention. Baker and colleagues[52] suggested a framework for developing and testing tobacco cessation interventions that divides the process of quitting into 4 phases: motivation, pre-cessation, cessation, and maintenance. The present study is focused on how best to get smokers into treatment and initiate cessation. Assessing outcomes at 1 month will best capture the immediate impact of OPT IN vs. OPT OUT on treatment utilization, quit attempts, and short term abstinence.[53,54] Moreover, assessing outcomes at 1 month accords with Joint Commission guidelines and UKanQuit follow-up practice for post-discharge follow-up of hospitalized smokers.[55] Perhaps most importantly, one month represents an important timeframe for hospitals, as hospitals with excessive 1-month readmission rates for selected diagnoses will receive decreased Medicare reimbursements.[56] Should our intervention prove effective, it could pave the way for future studies on the impact of smoking cessation on reduced 30-day readmission rates for specific diagnoses. Moreover, although we have powered the study to detect 1-month outcomes, our power will remain acceptable to detect differences and mediators of effects at month 6.

Clinical Trial Timeline and Procedures

The trial employs a post-test-only randomized study design among 1,000 inpatients with consent for extended data collection at month 1 and follow-up at month 6. It will be implemented in 4 stages over 4 ½ years (Table 1). Throughout the *Research Plan*, we address potential problems and alternative strategies after sections that pose potential risks in implementation. Below, we describe how we will alter the UKanQuit service's current practice to create the OPT OUT study arm and conduct the trial.

Table 1. Clinical Trial Timeline

Mo	Stage	Milestones (by end of year, unless otherwise stated)
6-23	1: Early Implementation	400 participants randomized<br>6-month outcome data collected on 200 participants<br>Manuscript on study design under review
24-39	2: Implementation	Baseline data collected on 800 participants<br>6-month data collected on 600 of participants<br>Data cleaned and prepared for analysis, ½ of participants
40-51	3: Early Analyses	Complete recruitment of final 200 participants by midyear<br>6-month data collection completed on all participants<br>Mscpt on baseline characteristics of participants published
52-60	4: Analysis/ Dissemination	All data cleaned and prepared for analysis<br>Conduct all outcome, mediator/moderator analyses<br>Manuscripts on study outcomes under review

Random selection for the trial. UKanQuit also has access via the EHR to a real-time, comprehensive list of all smokers in the hospital—the *tobacco use list*. For the purposes of the study, research staff will randomly select patients from the tobacco use list for the trial, and provide selected patients' names to UKanQuit staff for baseline assessment, randomization, and intervention. Random selection of participants serves 2 purposes. First, it ensures that we will test the change of default among a sample representative of all hospitalized smokers, which will enhance generalizability of the findings. Second, it ensures that smokers *not* seeking tobacco treatment will be included in the trial, which will enhance our ability to detect the effect of changing the default among smokers who have not requested, and who are not "ready", for treatment. Patients who requested or who have orders for tobacco treatment will be treated as usual by UKanQuit, and will only be included in the trial if they are randomly selected.

Eligibility and intake assessment. UKanQuit hospital staff will visit all randomly selected smokers. For all study patients, staff will assess and address patient comfort, provide brief advice to quit, and provide the 8-page pamphlet to all OPT OUT patients and to OPT IN patients who state their willingness to quit smoking. A 2-page brochure will be provided to all OPT IN patients who state they are not willing to quit smoking when they leave the hospital. UKanQuit staff will then assess eligibility and collect intake data. Study eligibility criteria are minimal: 1) be age 18 or over, 2) speak English or Spanish, 3) have access to a telephone or mobile phone, 4) not be

currently pregnant or breast feeding, 5) have no significant co-morbidity that precludes participation (i.e. acute, life-threatening illness, communication barriers such as a tracheal tube placement, or altered mental status such as dementia, or discharged to hospice or palliative care), 6) be a permanent resident of the state of Kansas or Missouri, 7) not currently prescribed or taking nicotine replacement therapy or varenicline during this hospitalization, 8) medically eligible to use nicotine replacement therapy (patient currently hospitalized with burns, acute myocardial infarction/STEMI, cardiac arrest, unstable angina, uncontrolled arrhythmia, stroke, peripheral arterial disease vascular surgery will not be eligible for inclusion), 9) patient not already seen by UKanQuit staff as part of the hospital based clinical service, 10) provided a secondary phone to ensure one month follow-up survey completion, 11) smoke one or more cigarettes on 25 out of the past 30 days 12) not taking medication to help in quitting smoking prior to admission, 13) not currently participating in a quit smoking program, 14) has been admitted to the hospital greater than three days 15) completed all eligibility questions 16) not in the process of being discharged and 17) already screened for eligibility greater than two times. Based on EQUIP data, we estimate that 8% of potential participants will be excluded due to age, language, or comorbidity. UKanQuit staff will record eligibility for the study on their clinic service tablet computer, along with standard service administrative data, which includes demographics, smoking characteristics including readiness to quit, and contact information for 1-month follow up. These administrative data will constitute baseline data for the clinical trial. The few patients who are not eligible will be provided UKanQuit services (as outlined in OPT IN procedures, below) but will not be enrolled into the trial.

Random allocation to study groups. A function will be programmed into the tablet intake form so that UKanQuit staff will select a key to randomize eligible smokers to either OPT OUT or OPT IN. UKanQuit staff will assist smokers to quit in accordance with the treatment to which patients are randomized.

OPT IN and OPT OUT intervention procedures—framing the default treatment. We have created draft language that constitutes the "choice architecture" for each study condition (Table 2). We have crafted these phrases to be short and simple, to enable UKanQuit staff to reliably use them. Based on the patients' group assignment, staff will frame the default and provide the appropriate intervention. OPT IN is the current protocol, using the same language, as UKanQuit staff use in their current procedures.

For OPT OUT language, we operationalize constructs thought to underpin the power of the default.[23] These terms: 1) signal the provider's positive attitude towards treatment, and 2) state that the hospital's *status quo* is to provide tobacco treatment. Using this OPT OUT language, UKanQuit staff will make it clear that they feel the patient should accept treatment, and that the hospital routinely treats all smokers.

Table 2. Choice Architecture, By Study Arm

**Components**	**Default: OPT OUT**	**Default: OPT IN**	
FRAME TREATMENT:	*"Because quitting is the best thing you can do for your health, KUMed provides free tobacco treatment for everyone who smokes."*	*"Quitting is the best thing you can do for your health. Are you ready to quit smoking in the next 30 days?"*<br>yes	no
FRAME INPATIENT COUNSELING:	*"Let's create a brief treatment plan that outlines your thoughts, feelings, and plans to treat your tobacco use"*	*"If you'd like, we can create a brief treatment plan that outlines your thoughts, feelings, and future plans about your tobacco use."*	Brief motivation: *'If it's ok with you, I'd like to talk with you about the risks of continuing to smoke and the roadblocks you're facing in trying to quit."*
FRAME REFERRAL TO OUTPATIENT COUNSELING:	*"We refer everyone to Kansas' free Tobacco Quitline—KanQuit."*	*"Would you like to participate in the free Kansas Tobacco Quitline?"*	
FRAME TAKE-HOME NICOTINE REPLACEMENT:	*"We send everyone who is medically eligible home with 2 weeks of free nicotine replacement."*	*"If you are medically eligible, would you like 2 weeks of free nicotine replacement?"*	

Inpatient counseling. For <u>all</u> patients in OPT OUT, staff will provide brief practical counseling and complete a treatment plan as outlined under UKanQuit current practice (*Appendix 3*). Staff provide a paper copy of the plan to the patient. For patients contacted over the telephone due to the COVID-19 pandemic, a copy of this plan will be mailed to the patient's home following discharge. For patients in OPT IN, staff will screen for readiness to quit. Patients who are ready to quit will be offered the same counseling and treatment planning as patients in

OPT OUT. Patients who are not ready will receive brief motivational intervention using the "5 Rs."

Post-discharge counseling. UKanQuit staff will enroll all OPT OUT patients into counseling for tobacco treatment after discharge. To OPT IN patients who are ready to quit, UKanQuit staff will offer counseling postdischarge. If the patient accepts, the enrollment will be made the same way as OPT OUT. Postdischarge telephone-based counseling will be provided by UKanQuit hospital staff. Patients who initially engage in counseling but at some point decide they no longer want any more post discharge counseling calls will be mailed a letter reminding them that they may contact us at any time if they decide they would like to reengage in counseling for their tobacco use. We will also remind the patient that we will be calling them one month post discharge to assess their experience with our program and their current tobacco use.

Post-discharge nicotine replacement therapy (NRT) "starter pack" and medication planning. All patients in OPT OUT will be provided with 14 days of either a) nicotine patches, b) nicotine mini-lozenges and/or c) nicotine gum, depending on contraindications, past history of success/failure, and personal preferences.[3] We opted to provide NRT because, in the most recent Cochrane meta-analysis of hospital interventions for smoking cessation, the addition of NRT significantly increased cessation rates over counseling alone, but addition of bupropion or varenicline did not improve post-discharge quit rates.[28] At the close of the session, UKanQuit staff will deliver the medication starter pack, with instructions for use, to the patients' bedside. During the COVID-19 pandemic when staff are unable to deliver medication to the patient's bedside, this medication will be mailed to the patient's home following discharge. If for any reason the resident, attending physicians, or the patient's floor pharmacist might have concerns regarding the patient using over the counter nicotine replacement medication upon discharge the medical team may contact the UKanQuit medical director, and present study co-investigator, Dr. Edward Ellerbeck, to discuss concerns. If it is determined the patient is not medically eligible for nicotine replacement medication the patient will be dis-enrolled in the clinical trial and treated as an UKanQuit patient. To OPT IN patients who are ready to quit, UKanQuit staff will offer nicotine replacement. If the patient accepts, the medications will be provided in the same way as OPT OUT. All patients provided with starter packs will also complete a pharmacotherapy guidance form with patients to select a long-term cessation medication and plan how they will obtain and fill prescriptions (if necessary) post-discharge. UKanQuit staff will then approach the patient's floor pharmacist to recommend a discharge prescription be ordered for the patient if medically acceptable to the patient's physician and medical team.

Design considerations: medications. We decided to provide starter packs and medication planning in order to help patients bridge the gap between hospital discharge and outpatient care. With the implementation of the Affordable Care Act, tobacco treatment is an Essential Health Benefit.[57] In addition, Kansas Medicaid covers all forms of first-line medication, and the national Pfizer Pharmacy Assistance Program provides varenicline and the nicotine inhaler free of charge. Last, nicotine replacement therapies are available over the counter at low cost. Consequently, some type of tobacco treatment medication should be covered for virtually all patients. The actual nature of these benefits, however, varies substantially across health plans, is constantly evolving, and requires some time for patients and providers to sort out. In order to reduce the impact of variable medication access on study outcomes, we elected to 'standardize' medication access by providing starter packs of NRT in both treatment arms, and helping patient plan for how they will obtain medications post-discharge. The medication planning procedures used here will be very similar to the pharmacotherapy guidance provided by Dr. Richter's staff as part of a study examining the impact of telemedicine-delivered smoking cessation counseling (R01-HL08764301).[58]

Quitline services. All patients in OPT OUT and patients ready to quit in OPT IN will be enrolled in post-discharge counseling. Participants who accept enrollment into counseling services will receive up to 4 proactive counseling calls . Each call is designed to provide practical counseling to help participants develop problem-solving and coping skills, secure social support, and design a plan for successful cessation and long-term abstinence. Initial calls last approximately 30 minutes and follow-up calls last on average 15 minutes. Once participants quit, UKanQuit counselors review high-risk situations, coping skills, and stress management to prevent relapse. When participants slip, counselors troubleshoot relapse situations and encourage smokers to quit again. We will create custom computer systems to store data for all callers, including number of attempts to reach the smoker, number of calls completed, and duration of calls. All counseling calls will be recorded for quality control as per the UKanQuit treatment service protocol. Patients will be informed at the beginning of the call that the session will

be recorded. Patients will have the option to opt out of having the session recorded. A random selection of sessions will be reviewed for quality. Peer group supervision and one on one supervision will be conducted by listening to a 1-2 recordings each week to improve the overall counseling. Recordings will be stored in a secure, access limited location on the Share drive. Recordings will be deleted on an ongoing monthly basis.

*Month 1 UKanQuit call for service data collection, informed consent, and reimbursement***.** In accordance with current service protocol, UKanQuit staff will call all study participants at 1 month-post randomization to assess UKanQuit service outcomes including smoking status, quit attempts, counseling utilization, medication use, and other factors related to quitting (see *Table 3, Core Study Measures*). At the close of the call, UKanQuit staff will verbally debrief patients on the clinical trial, invite patients to consent to the first in-hospital phase of the trial (Phase 1) and invite patients to participate in extended follow up surveys (Phase 2). UKanQuit staff will transfer consenting patients to research staff for data collection. Trained research assistants blinded to treatment allocation will conduct extended assessments at one month and 6 months following randomization. Additional details on consent and reimbursement are provided under **Protection of Human Subjects**, below.

Patients will be reimbursed $10 at baseline for time the intake assessment requires of patients. Patients who participate and complete Phase 1 will be reimbursed an additional $25 at month one for activities completed regardless if they consent to Phase 1 allowing us to utilize the data collected as a part of our clinical trial. Study staff will reimburse patients who consent to participating in Phase 2 with an additional $25. All reimbursements will be via reloadable debit cards. The debit cards utilize the MasterCard payment system and are accepted at virtually every institution that accepts a credit card. Issuing debit cards and providing a minimal payment of $10 to patients at baseline will enhance patient's willingness to provide their social security number (in person) versus providing it one-month post discharge over the telephone. Credibility will also be enhanced when UKanQuit staff call to complete a survey one-month post discharge, since this will allow the staff to refer to the debit card number provided in the hospital. Providing the debit card at baseline will also build patient confidence that UKanQuit are affiliated with KUMC and aren't trying to gain access to their social security number illegally. Participants will be reimbursed $25 for each survey completed. Participants who indicate they have quit smoking participating in Phase 1 or Phase 2 of the clinical trial will be asked to provide either a salivary sample for cotinine testing or a carbon monoxide breath test to verify their quit status. During the COVID-19 pandemic carbon monoxide testing in-person was discontinued. Participants who provide verification will be reimbursed $150.

Potential Problems & Alternative Strategies

Hard to reach patients and patients who choose to opt out. As with standard inpatient counseling, staff may initially find patients occupied with hospital procedures, but will return when patients are free to participate in counseling. Similarly, at times counseling sessions are interrupted, if the hospital procedure is brief the counselors will wait and resume the session afterwards. If it is lengthy the counselor will contact the patient at a later time. For patients in the OPT OUT arm who choose to opt out from any component of care (such as post discharge counseling, or medications), UKanQuit staff will encourage participants to use the contact information in the UKanQuit treatment pamphlet to obtain counseling and/or medications as soon as possible.

Patients who visit the hospital frequently. Data from EQUIP suggests 20% of smokers will be re-admitted within 6 months of discharge. To adhere to current treatment guidelines[55], all patients readmitted within 6 months will receive another bedside or telephone consult from UKanQuit. Patients will remain within the groups to which they were initially assigned, and they will receive the treatment to which they were originally assigned. Consequently, some patients in each study arm will receive multiple interventions. This should occur in real-world practice. Sensitivity analyses will be conducted to assess the impact of multiple hospitalizations on outcome.

Subject follow-up. Our ability to contact and recruit a high percentage (80% or more) of patients seen at baseline is essential to the success of this study. EQUIP has been able to retain 89% of study participants at one month, and 85% of participants at 6 months, following randomization. We are confident we will be able to recruit 80% of eligible inpatients into our trial for the following reasons: 1) we will collect multiple methods for contacting patients after discharge, including phone numbers, emails, and social network contacts (Facebook pages); 2) we will offer $10 at baseline and a $25 gift card for each assessment point, 3) patients will be consenting to very minimal research participation, consisting of survey items and a mailed salivary cotinine or anabasine sample or a carbon

monoxide sample among those who report quitting (during the COVID-19 pandemic carbon monoxide testing was discontinued); 3) patients will be asked for verbal consent, to reduce attrition from mailed consent procedures; 4) we will inform patients after the counseling session (in the hospital or over the telephone) that we will be calling them at one month post discharge to evaluate our services and for completing this call we will reimburse them $25; 5) we will reimburse smokers who complete Phase 1 regardless if they consent to participate in Phase 1 or 2 of the clinical trial; 6) we will mail participants reminder letters and postcards about the upcoming one month survey; 7) we will obtain written permission from patients to send text messages reminding them we will be calling them in 1-2 days at one month post discharge to evaluate our services. Text messages will be sent to patient's cell phones who gave permission. Text messages will be sent via the UKanQuit service cell phone which is password protected and is used by treatment service team only. Finally, 8) we will provide home visits for participants who need them, except during the COVID-19 pandemic.

Attrition due to failure to provide consent. We expect to see minimal and equivalent attrition in both study arms. Due to the large sample size and random allocation, there should be equal proportions of patients who are motivated to quit in each study arm. If, therefore, patients who are less motivated to quit decline participation at month 1, they should withdraw in equal proportions across groups. Hence, any bias that will be introduced by attrition at month 1 should affect the overall study findings, not differences between the groups. We will minimize attrition by offering participants to consent to Phase 1 of the study only if they are not willing to consent to Phase 2, keeping Phase 2 activities to a minimum, and reimbursing participants for their participation in Phase 2. As described under **Preliminary Studies**, we typically have very low subject attrition (11% at 1-month) in our hospital studies. In order to examine whether there is any differential attrition between groups, we will compare the composition of each study group before and after the 1-month follow up. Should we find significant differences between the pre- and post-follow up groups, we will conduct post-hoc sensitivity analyses to assess the impact of attrition on findings.

Project Measures (Table 3)

Data reside in the UKanQuit service database, which our database manager, Niaman Nazir, manages. Among participants who provide consent, we will conduct additional surveys at months 1 and 6 and collect data on counseling participation.

Tobacco abstinence. Outcome measures are adapted from the Society for Research on Nicotine and Tobacco's Workgroup on Abstinence Measures and Workgroup on Biochemical Verification.[59,60] Our primary endpoint is 7-day, self-reported and verified cigarette abstinence at 1 month after randomization. In accordance with common analytic procedures for tobacco treatment trials, patients who do not consent to follow up, and those not reached or not verified, will be counted as smokers.

Verification of abstinence. We will use either mailed salivary cotinine or anabasine or in-person carbon monoxide (CO) testing to confirm smoking status. During the COVID-19 pandemic carbon monoxide testing was discontinued. Using this combination, we have verified the proportions of participants who self-reported abstinence and verified by cotinine and CO were 94% and 3.0% respectively. Participants who report 7-day point prevalence abstinence, and who are not taking nicotine replacement, will be asked to provide a salivary cotinine sample. Cotinine is the measure of choice because of its sensitivity and specificity.[60] We will use a cut-point of ≤10 ng/ml to differentiate smokers from nonsmokers.[61] During the COVID-19 pandemic when carbon monoxide testing is discontinued, we will use mailed salivary anabasine testing for participants who report not having smoked in the past 7 days, but who report use of nicotine replacement, electronic cigarettes or other tobacco products. The cut-point for anabasine will be <1 ng/ml to differentiate smokers from nonsmokers. Samples will be stored in a -20° freezer until laboratory analysis. Participants who are still using nicotine replacement, or who refuse salivary cotinine, will be verified via CO, except as described above. Those with ≤10 ppm will be considered abstinent.

**Table 3. Core Study Measures**	**Baseline**	**Mo. 1/6**
**UKanQuit Service Measures (all participants)**		
Demographics: age, gender, race	✓	
Readiness to quit, craving/withdrawal	✓	✓
# Cigarettes per day (cpd); time to first cigarette	✓	✓
Motivation/confidence quit/stay quit	✓	✓
7-day point-prevalence abstinence		✓
# of quit attempts since enrollment		✓
Medication use/adherence		✓
**Research Assistant collected measures (consenting participants)**		
Biochemical quit verification		✓
Default constructs (perceived status quo, implied recommendation, perceived paternalism)		✓
Length of hospital stay (for index visit)	✓	
Reason for hospitalization (index visit)	✓	
Re-hospitalization w/in 30 days of discharge		✓
Outpatient counseling use/adherence		✓
5-Trial Adjusting Delay Discounting Task		✓
**Cost measures (consenting participants)**		
Counseling (calculated from UKQ/Alere data)		✓
NRT (calculated from patient self-reported use)		✓

Secondary outcomes, mediators, and moderators. Counseling data will be collected throughout the trial, which will be summarized as 'total counseling time' for analyses. We will assess the type, the dose, and the number of days medication was used via the method of Williams et al.[65,66] This will be summarized as "number of days of medication use" for analyses. Default-related variables are derived from the literature on choice theory and include smokers' perceptions of provider attitudes toward tobacco treatment (implied recommendation), smokers perceptions of the degree to which their provider recommends tobacco treatment (implied recommendation), perceptions of the "status quo" for hospital tobacco treatment (status quo bias), and perceptions of paternalistic treatment by UKanQuit staff.[17,23,67] We will develop several new survey questions to measure aspects of changing the default. In order to do so, we will adapt 2 validated surveys—the Working Alliance Inventory (WAI), and the MacArthur Admission Experience Survey. We will slightly change the language in each, and reduce the number of items in the WAI. In order to test the "usability" of these adapted surveys, we will administer these to an anonymous sample. Amazon M*Turk is a crowdsourcing software designed by Amazon and is regarded as a valid method for research in social and behavioral sciences (e.g. Mason & Suri, 2012; Buhrmester, Kwang, & Gosling, 2011). In order to test the readability and psychometrics of our modified survey items, we will post a request for completion of a questionnaire on M*Turk. Written informed consent will not be collected, as the surveys are about very low-risk information and we will not collect any respondent identifiers.

We will request that MTurk collect responses from 100 individuals, for a 5-minute survey, for $1.00 per respondent. Eligibility criteria include Turkers who are 18 years or older, have smoked on a daily basis within the past 6 months, and had a health care provider advise them to quit in the past 6 months. Once this order is completed, MTurk will make available a spreadsheet for download. We will download the data and factor analyze data to see if we will be able to eliminate extraneous items from our proposed scales. The final questions will be utilized for the clinical trial and will be included as part of the one month survey.

We will use MTurk to also validate a new measure, Tobacco Working Alliance Inventory (TWAI), against the established published measure the Working Alliance Inventory (WAI). This will establish the credibility of the TWAI as a shorter measure of the "gold standard" WAI. We will request that MTurk collect responses from 100 individuals, for a 5-minute survey, for $1.00 per respondent. Eligibility criteria include Turkers who are 18

years or older, have smoked on a daily basis within the past 6 months, and had a health care provider advise them to quit in the past 6 months. Once this order is completed, MTurk will make available a spreadsheet for download. We will download the data and factor analyze it to further assess the validity of our adapted instrument, the TWAI.

Additionally participants will perform a one minute 5-Trial Adjusting Delay Discounting Task during the baseline, month 1 and 6 surveys (Koffarnus and Bickel, 2014). Participants will be read in person (baseline) or over the telephone (follow-up) a series of discounting tasks. Responses on these tasks will be recorded. Individuals who discount delayed rewards at a high rate may be more likely to engage in treatment.

Intervention costs. We will prospectively track variable intervention costs. Costs will include inpatient counselor services, postdischarge counselor time, and initial pharmacotherapy dispensed at baseline. During the 6-month follow-up call, we will ask participants to recall their use of pharmacotherapy after the initial supply. Personnel time will be valued at Bureau of Labor Statistics (www.bls.gov) wages plus benefits for an appropriately trained health promotion professional. Pharmacotherapy costs will be based upon retail prices estimated from on-line pharmacy websites, e.g., www.drugstore.com during the study. Intervention costs will be tracked as they are incurred. We will exclude research costs. Given that all costs are short-term (<6 months), we will not discount either costs or benefits.

Fidelity monitoring. Fidelity to components will be assessed by in-person fidelity assessment checks during hospital consults and by digitally recording inpatient counseling sessions. To assess the quality of the intervention and control conditions, we will assess the degree to which UKanQuit staff accurately: 1) identify eligible/ineligible participants; 2) provide brief advice and the study pamphlet; 4) randomize patients and perform the appropriate intervention for OPT OUT and OPT IN; 5) address post-discharge medications; and 6) provide information regarding the post discharge counseling to the appropriate patients. Digitally recording will only be conducted on patients who provide written consent for the audio recording of the counseling session. Recording will allow for increased fidelity monitoring of patient counseling sessions. Recordings will be stored in a secure, limited access location on the share drive. The sessions will be coded and entered into the fidelity database. Recordings will also be used during group and one on one supervision sessions to improve the overall quality of the counseling sessions. Recordings will be permanently deleted at the conclusion of the clinical trial. Data on fidelity will be entered into a database and reported back to hospital staff on a monthly basis to encourage adherence to protocols.

Data Management

Data management will follow procedures developed for EQUIP. UKanQuit service data, and survey data collected by research assistants, will be directly entered via tablet into REDCap. Project Director Mussulman will coordinate data retrieval from the EHR. Data manager Mr. Nazir will conduct initial data cleaning, identifying and tagging any crossovers, conversion into proper format for data analysis, and recoding using standard operating procedures. All data will be imported into SAS for study analyses. Cleaning and management routines (e.g. conversion of birth dates to ages, logical checks for continuous variables, compliance with skip patterns, missing data codes) will be conducted using SAS.

Data Analysis: Overview of Hypotheses and Analyses (Table 4)

The overall study design is a posttest only design with random assignment to groups. We will conduct process, outcome, mediation, and cost analyses. Prior to initiating outcome analyses of quantitative data, we will compare baseline data across groups to evaluate whether random allocation achieved equivalent groups. Bayesian analysis (see *Statistical Model*, below) will answer our main outcome. After verifying adequate SEM fit to the data, i.e., that CFI >.9 and RMSEA < .8, we will use SEM to perform classic mediation analysis using the strategy outlined by Baron & Kenney[68] (see Figure 1, under *Significance*, for theoretical model). When conducting the final analysis we will exclude the following: participants who refused consent, patients who died or are incarcerated. We will test whether there are any systematic differences between the enrolled and non-enrolled population by comparing the demographic and tobacco use patterns of all non-enrolled participants (unable to reach, deceased, incarcerated, refused consent) to those who enrolled (consented) at baseline. This comparison will strengthen considerably the scientific merit of the study by enabling reviewers and readers to judge how representative our study population was to all hospitalized smokers treated.

Table 4. Study hypotheses, measures, and analytic strategy

Purpose	Variables	Analytic Strategy
Hypothesis 1: Compared to **OPT IN**, significantly more in **OPT OUT** will participate in counseling, use cessation medications, and be abstinent from smoking	Abstinence: Treatment condition and 1-month 7-day point prev. abstinence	Bayesian analysis
	Counseling: Treatment condition and total counseling time by 1 month	T-test
	Medication: Treatment condition and number of days of medication use	T-test
Hypothesis 2: Significantly more smokers in **OPT OUT** will be abstinent from smoking, and mediation analyses will partially or fully explain the effects.	Treatment condition and 6-month and<br>-7-day point prev. abstinence<br>-Default variables<br>-Counseling/medication use	Structural equation modeling with a logistic outcome
Hypothesis 3: **OPT OUT** will be more costly but also more effective than **OPT IN**	Treatment condition and 1-month<br>-7-day point prev. abstinence<br>-Variable costs	Incremental cost/quit

Data Analysis: Bayesian Study Design, Outcome Analyses, and Cost Effectiveness

We will perform a prospective randomized comparative effectiveness *Bayesian adaptive design study*.[69] This approach is a highly efficient and ethical strategy for comparative effectiveness clinical trials design, because it allocates more patients to effective treatments and can answer the research question earlier than conventional designs.[70] In Bayesian adaptive designs, one primary endpoint is used to drive the adaptive randomization. This endpoint is compared across study groups periodically, and more patients are randomized to the stronger arm, until a predetermined probability that one arm has "maximum utility" is reached, which signals the end of the comparative trial. Our endpoint is the percentage of patients who quit smoking at 1-month (4 weeks) post-study randomization. We will perform our first planned interim analyses when we have randomized 400 patients. Based on the 400 randomized patients, the first interim analysis final set will be sub-selected via patients consenting to be enrolled or patient unable to reach for the 1-month survey, and patients with 1-month survey window closed. The arm that appears to be performing the best will get more participants allocated to it in the subsequent randomization period. A new adaptive randomization structure will be updated every 13 weeks, using up-to-date outcome data, until a) trial meets early success or b) randomize all 1,000 participants. All outcome analyses will use an intent-to-treat approach, in which all participants will be included in the group to which they were originally assigned.

Virtual participant response. In accordance with guidelines for adaptive design power analyses[69], we assumed several virtual (or "pretend") responses to determine the power, sample size and time (duration) needed for our study. We created several scenarios for quit rates using three assumptions (Table 5). One virtual response is the 'expected' quit rates, another is 'small but unlikely' quit rates, and the third is 'no differences' in quit rates.

Accrual (enrollment) patterns. Accrual patterns refer to how rapidly we enroll patients in the trial. These are important to Bayesian adaptive designs for determining the length of the trial. Based on accrual patterns for EQUIP and other hospital studies conducted by Drs. Richter and Ellerbeck, we assume that the accrual patterns will follow a Poisson distribution with an average of 6.7 patients per week.

Table 5. Virtual response patterns for quit rate endpoint

	OPT IN	OPT OUT	
		Efficacy	
No differences	15.7%	15.7%	Both have equal quit rates
Small but unlikely	15.7%	20.0%	Opt-Out is moderately better
Expected	15.7%	25.2%	Opt-Out is better at expected differences

Statistical model. For this study the primary endpoint is modeled $S_{Qj}|n_j \sim \text{Bino}(n_j, \theta^Q_j)$ quitting. In addition, we provide "weakly informative" priors, $\text{logit}(\theta^Q_j) \sim N(0,100^2)$. Using the endpoint data and the prior probabilities, we then use Markov Chain Monte Carlo computations to obtain the Bayesian posterior distributions for the endpoint (i.e., quitting.) We will stop the randomizing into the comparative trial if the probability of a study arm having best utility is greater than 0.9925 at both 1-month AND 6-months. The arm (or drug) having the maximum quit rate is $M_T=\max(\theta^Q_1, \theta^Q_2)$. The stopping rule is mathematically $P(\theta^Q_1 > \theta^Q_2) > .9925$ or $P(\theta^Q_1 < \theta^Q_2) > .9925$), this would take place both at 1-month AND 6-month endpoints. If a best arm is not identified after 500 patients randomized, this procedure and accrual will continue until a best arm is identified or we randomize all 1000 patients. Should

we reach our stopping rule before 1,000 patients randomized, we will continue to recruit patients, but we will stop randomization and recruit the remaining patients into the more effective study arm. We will do so because this is the first trial to experimentally test the impact of changing a treatment default. We believe it is important to maximize our cases to enable us to conduct mediation analyses that will determine the mechanisms that underlie the impact of treatment defaults.

Adaptive Randomization: allocation. After the best utility probability is evaluated the next round of patients are randomized using a formula, which is V^*_j=sqrt($P(\theta^Q_j > \theta^Q_{j'})$Var($\theta^Q_j$)/($n_j$+1)) and θ^Q_j and $\theta^Q_{j'}$ are the utility parameter (i.e. smoking quitting rate parameter) of the two arms at 1-month only, that takes advantage of the information gained from our analyses up to that point. The newly enrolled patient allocated to the jth arm is proportional to V^*_j=sqrt($P(\theta^Q_j > \theta^Q_{j'})$Var($\theta^Q_i$)/($n_{jT}$+1)). This type of allocation tends to have more desirable properties then simply using Pr($M_{jT}= \theta^Q_1$). In other words, using this approach will allow us to assign more patients to the most promising arm, and fewer patients to the least. Regardless of when the probability cutpoint is reached, we will confirm this finding with a subsequent analysis and evaluation (>.99), which can be at 1-month OR 6-month endpoints, after all data from patients are obtained, as some will still be actively in the study when the early success criterion is identified.

Power, sample size, and trial duration. We performed three sets of trial simulations based on the various combinations of quit rates endpoints for both 1-month and 6-months (Table 5). Each set involved many trial simulations that identified power (the probability of success) in two scenarios—one for early success (i.e., being able to stop randomization early) and one for late success (i.e., upon randomizing all 1000 patients). While two of these combinations are very unlikely to occur, we included all scenarios. <u>First</u>, under the 'expected' quit rates at 1-month and 'expected' at 6-months, we estimated (identified) that 75% of the simulated trials had early success, 24% late success, and only 1% had incomplete results. Thus this scenario had 99% power. The average sample size of this trial scenario was 789 patients with more than half (546) in the better OPT-OUT arm. The average length of these simulated trials was 145 weeks. <u>Second</u>**,** if there is 'expected' quit rates at 1-month and 'small but unlikely' quit rates at 6-months, we estimated (identified) that 23% of the simulated trials had early success, 68% had late success, and 9% had incomplete results. This trial scenario had 91% power and the sample size of this trial scenario was on average 947 with more than half (696) in the better OPT-OUT arm. The average length of this trial scenario was 167 weeks. <u>Third</u>, we examined the scenario that serves as our null hypothesis <u>(no differences) at both 1-month and 6-months.</u> In this scenario there are no differences in quit rates among the arms. The extent to which this scenario is "successful" actually reflects our Type I error rate. For this scenario, we estimated (identified) that 0% of the simulated trials had early success, 5% late success. Thus this trial scenario produced an appropriate expected Type I error (α=.05,). The sample size of this scenario on average was 1000 patients, with half (500) in the OPT OUT arm. The average length of the trials under this scenario was 175 weeks— approximately 3 years of recruitment. Hence, our sample size of 1,000, in 3 years of recruitment, provides ample time and participants to identify project outcomes under all 3 scenarios.

Cost analyses for Hypothesis 3. We will conduct a cost-effectiveness analysis to explicitly document the relative costs and benefits of OPT OUT versus OPT IN. This analysis will be conducted in collaboration with Dr. Theresa Shireman at Brown University. Dr. Nazir and Dr. Shireman will manage the cost effectiveness analysis. Dr. Nazir will send Dr. Shireman de-identified data sets through secure electronic channels to ensure the protection of the confidentiality of participants. Our cost analytic framework generally follows the guidelines adopted by the Centers for Disease Control (CDC) in accordance with the consensus Panel on Cost-Effectiveness in Health and Medicine.[71-73] We will divide the analysis into two components: first, intervention only costs, and second, intervention plus short-term (<=6 months) costs post-discharge. The primary cost-effectiveness analysis will be set up as an incremental cost-effectiveness ratio (ICER). We anticipate that OPT OUT will be more costly and more effective than OPT IN. Incremental cost-effectiveness analysis identifies the marginal benefit of switching from one intervention to the other and is the ratio of the difference in costs divided by the difference in effectiveness between the two treatment options. The outcome assessed will be biochemically verified 7-day point prevalence abstinence. The ICER will indicate the added cost per additional quitter OPT OUT versus OPT IN, a metric that will allow comparisons to other smoking cessation economic studies. In designing these analyses, we considered using a societal perspective, as recommended by current national guidelines.[71] The societal perspective, however, requires quality-adjusted life years (QALYs) as the denominator. Since this is a short-term study, we decided against attempting to estimate changes in QALYs, and focus instead on cost per

quit.

The data derived for this cost analysis come from several clinical trials based in Kansas.[35,49] In sensitivity analyses, we will adjust wages rates upwards to the national average. In order be able to generalize our findings from this one clinical trial to other populations, we will explore how the variation in counseling time and effectiveness influence the relative cost-effectiveness of the treatment strategies. Our analyses will vary time and effectiveness until breakeven points are achieved between the treatment options.

PROTECTION OF HUMAN SUBJECTS

Risks To The Subjects

Human Subjects Involvement and Characteristics. The primary research interest in this study is to determine the population impact of changing the default for tobacco treatment by examining the impact of providing all smokers with cessation medications and counseling unless they refuse it (OPT OUT) versus current practice—screening for readiness and only offering cessation medications and counseling to smokers who say they are ready to quit (OPT IN).

The study is a randomized clinical trial with delayed verbal consent. It is conducted in 2 Phases. In Phase 1, hospitalized smokers who are admitted to the University of Kansas Hospital (KUMed) will be recruited. Participants must be 1) be age 18 or over, 2) speak English or Spanish, 3) have access to a telephone or mobile phone, 4) not be currently pregnant, 5) have no significant co-morbidity that precludes participation (i.e. acute, life-threatening illness, communication barriers such as a tracheal tube placement, or altered mental status such as dementia, or discharged to hospice or palliative care), 6) be a permanent resident of the state of Kansas or Missouri, 7) not currently prescribed or taking nicotine replacement therapy or varenicline during this hospitalization, 8) medically eligible to use nicotine replacement therapy (patient currently hospitalized with burns, acute myocardial infarction/STEMI, cardiac arrest, unstable angina, uncontrolled arrhythmia, stroke, peripheral arterial disease vascular surgery will not be eligible for inclusion), 9) patient not already seen by UKanQuit staff as part of the hospital based clinical service, 10) provided a secondary phone contact to ensure one month follow-up survey completion, 11) smoke one or more cigarettes on 25 out of the past 30 days, 12) not currently taking medication to help in quitting smoking, 13) not currently participating in a quit smoking program, 14) has been admitted to the hospital greater than three days 15) completed all eligibility questions 16) not in the process of being discharged and 17) already screened for eligibility greater than two times. Patients will be randomized into study arms, receive the intervention to which they were assigned, followed up at month 1 by clinical staff. At this point, clinical staff will debrief patients regarding their inclusion in Phase 1 of the clinical trial. Staff will ask patients for delayed consent to retain their data in Phase 1 of the trial and will be invited to participate in Phase 2 of the trial. Patients who refuse will be asked if we have their consent to retain the data collected thus far in Phase 1 while not participating in Phase 2. Patients who consent to Phase 1 only but have self reported quitting smoking will be asked if they are willing to provide a saliva or carbon monoxide sample to verify their quitting. During the COVID-19 pandemic carbon monoxide testing will be discontinued. Patients who refuse all aspects of the clinical trial (Phase 1 and 2) will removed from the trial.

Phase 2 consists of two follow-up assessments. We provide an extended rationale for this design under *Recruitment and Informed Consent*, below. The study design has some similarities to a Zelen study, in which patients assigned to experimental conditions are asked to provide informed consent after randomization.[74]

The study involves testing alternative methods of engaging hospitalized smokers in treatment. After patients are admitted to the hospital, UKanQuit hospital staff will identify every smoker via the electronic health record (EHR), randomly select the daily quota of study participants, and visit the selected patients. At the bedside, or during the COVID-19 pandemic when staff were unable to meet at the bedside then over the telephone. UKanQuit staff will briefly advise all smokers to quit, provide an 8-page pamphlet on cessation resources, and use a tablet computer to randomly assign patients to one of 2 study arms: OPT OUT or OPT IN. UKanQuit staff will administer the appropriate intervention. UKanQuit staff will collect contact information for their 1-month post discharge

phone assessment, which is standard practice for the service in accordance with Joint Commission follow-up recommendations. After discharge, UKanQuit staff will contact all smokers seen. They will collect month 1 service data, describe the procedures for the research study, collect verbal consent and transfer interested patients to research staff who will conduct an extended month 1 survey. Consenting patients who report smoking abstinence at month 1 will be asked to provide a salivary cotinine or anabasine sample or carbon monoxide sample for verification of smoking status. Study research staff will conduct a similar assessment at month 6 among consenting patients. Outcome measures and analyses include biochemically -verified 7-day point prevalence abstinence at 1 and 6 months, quitline and medication utilization, assessment of mediators, and cost-effectiveness analyses. We estimate 1,000 participants, 500 in each arm, will be required to detect the expected treatment effect.

Sources of Materials: Intervention Materials. All patient education materials and surveys will be approved by human subjects prior to study implementation. UKanQuit staff will provide an 8-page pamphlet to all OPT OUT patients and to OPT IN patients who state their willingness to quit smoking. A 2-page brochure will be provided to all OPT IN patients who state they are not willing to quit smoking when they leave the hospital. (*Appendix 1*). Staff will complete a 1-page treatment plan with all patients in OPT OUT and patients ready to quit in OPT IN.

Sources of Materials: Study Data. The study will gather data from 3 separate sources: 1) UKanQuit service data for intake (baseline) data and 1-month outcome data; 2) Extended follow-up survey data collected by the study team; 3) Post-discharge counseling adherence data.

UKanQuit service data. These are clinical data that are collected at the time of randomization by UKanQuit staff either in person or, during the COVID-19 pandemic, over the telephone. These data are also collected at one month following inpatient bedside or telephone counseling. These data constitute our baseline and main outcome data.

Extended Follow-up Survey Data. These are survey data and biochemical verification data collected by research assistants among patients who consent to participating in research follow up at one month following bedside or telephone counseling. These data augment our main outcome data, as only patients who consent to follow up and provide verification of abstinence will be considered quitters.

Post-Discharge Counseling Data. These data include whether patients enrolled in post-discharge counseling services and the amount of time patients spent in counseling. Post-discharge counseling data from patients who consent to Phase 2 only will be collected for merging with study data.

Potential Risks. Risks for participating in the study are minimal and include those associated with the inconvenience of completion of several questionnaires and interviews, telephone follow-up assessments, telephone counseling sessions, and taking over-the-counter NRT. Saliva samples will be collected and analyzed from consenting participants who report abstinence at 1 and 6 months. No data are collected that would put participants at risk for criminal or civil penalties. Alternatives to participating in the study are to quit "cold turkey" (without assistance), use other smoking cessation programs, purchase other NRT from their pharmacy, obtain a prescription for bupropion or other smoking cessation products from their physician, or continue to smoke.

Adequacy of Protection Against Risks

Recruitment and Verbal Informed Consent. We will recruit 1000 participants on-site at KUMed. Our Human Subjects Committee has permitted us to alter typical consent procedures based on federal regulations for the protection of human research subjects (45 CFR 46): 1) the research involves no more than minimal risk to the subjects; 2) the alteration will not adversely affect the rights and welfare of the subjects; and 3) the research could not practicably be carried out without the alteration. The hospital approved the study approach because the control study condition (**OPT IN**) is in accordance with current treatment guidelines and is exactly what occurs in the hospital today—the **OPT OUT** condition is much more proactive (see *Letter of Support from Christopher Ruder, Vice President of Patient Care Services*). Hence, regardless of which condition a patient is assigned to, none will receive less care by participating in the study than they would if they were not participating in the study.

The difference between the study arms consists solely of the proportion of patients that may engage in evidence-based care. Because the treatment associated with smoking cessation is extremely safe, the risks are minimal.

Thought leaders in clinical ethics suggest that streamlined consent is appropriate for this type of trial. Faden, Beauchamp, & Kass assert that informed consent is not always necessary for randomized comparative effectiveness research (CER) trials[75,76], because many CER trials present minimal risks but the potential effects of the research on patient welfare is immense. Streamlined consent is acceptable for research in which patients' rights and dignity are respected, clinician's judgment is followed, patients receive optimal care, and non-clinical risks are minimal. Streamlined consent could facilitate research in areas where patients experience medical errors and mismanagement because research to correct these problems is unduly burdensome to conduct. The present application is a good candidate for streamlined consent because the risks are minimal, the potential payoff is large, and the need to include patients at all levels of motivation is paramount, but would be very difficult to achieve if written consent were sought prior to randomization.

The design is a hospital-based clinical trial with delayed verbal consent for participation and follow up. This feature of the design should facilitate enrolling more smokers in extended follow up and enhancing external validity, as 2 reimbursed follow up calls constitute the extent of smokers' active research participation. We use this design because this study tests the effects of providing treatment to all smokers; therefore, all smokers must be candidates for the trial. We are able to integrate the OPT OUT condition into an ongoing clinical service, which permits us to collect baseline and month 1 main outcome data prior to consent—as a part of clinical care. Our hospital tobacco service follows Joint Commission guidelines, which recommend that smokers receive an assessment within 1 month of hospital discharge. Patients whom we are not able to reach will be counted as smokers, in accordance with standard analytic procedures for tobacco treatment trials.

One month following inpatient treatment, UKanQuit staff involved in patients clinical care will contact patients to debrief on the clinical trial and collect verbal consent (See **Verbal Debrief and Consent**, below). In the consent process, UKanQuit staff will 1) debrief patients on their participation in Phase 1 of the clinical trial, 2) collect consent for retaining data in Phase 1 of the trial, 3) collect consent for participating in Phase 2 of the trial.

Issues covered in the verbal consent procedures include the reason for collecting delayed consent, a description of study procedures, the time involved, the right to withdraw at any time without penalty, procedures used to protect participant confidentiality, data collected in the study and the use of data, reimbursement, and potential benefits and risks of participating in the study. The verbal debrief and consent document has been approved by our Human Subjects Committee. The data that we collect in follow up will permit analyses of mediators of outcomes, long term quit rates and cost analyses (Aims 2 and 3).

Reimbursement. Patients who are randomized and complete the baseline phase of treatment will be reimbursed $10 for their time. Patients who participate in Phase 1 but do not consent will be reimbursed $25 for their time completing the Phase 1 assessment. Patients who participate in Phase 1 and not consent to Phase 1 only will also be reimbursed $25 for their time completing the Phase 1 assessment. Study staff will reimburse patients via reloadable debit cards. The debit cards utilize the MasterCard payment system and are accepted at institutions that accept credit cards. Following consent to Phase 1 and Phase 2 and participation in the Phase 2, month 1 follow up survey, staff will mail participants a card pre-loaded with $50. Participants who completed Phase 1 but do not consent will be eligible to receive reimbursement if they provide the required information (name, social security number, etc.) to receive a pre-loaded debit card in the mail from UKanQuit staff. Participants who completed Phase 1 and consent to Phase 1 only will also be eligible to receive reimbursement if they provide the required information (name, social security number, etc.) to receive a pre-loaded debit card in the mail from UKanQuit staff. Participants will be reimbursed $25 for each follow-up survey completed and $150 for each salivary cotinine sample returned or Carbon Monoxide measurement provided, where applicable.

Protection Against Risk: Bayesian design. The Bayesian adaptive design sequentially assigns patients to the stronger treatment arm, and randomization will stop as soon as the more effective study arm is identified. Hence, non-consenting participants will only be involved in a randomized trial so long as we do not know which study arm is the most effective. The study focuses on the manner in which patients are engaged in treatment. The actual treatment is the same across both groups. All participants are smokers, all treatment components are

indicated for all smokers, and they are the treatment components employed by UKanQuit in its current clinical practice.

Protection Against Risk: Non-Consenting Participants. Participants who refuse consent to Phase 1 will be entirely removed from the trial, with one exception; to use de-identified data from participants who did not consent and add their data with those who we were unable to reach or who deceased at one-month follow-up to compare the demographic and tobacco use patterns at baseline of all non-enrolled participants to those who enrolled. Participants who consent to Phase 1, but who refuse consent to Phase 2 will be included in Phase 1 but excluded from Phase 2 of the trial. Data from patients we are unable to reach at one month will be de-identified and included in data analyses.

Protection Against Risk: Consenting Participants. For consenting participants, we will also use data from the post-discharge counseling sessions. Salivary cotinine samples are non-invasive; samples will be labeled with participants' study ID numbers, rather than names, to protect participants' privacy. When collecting carbon monoxide verification of smoking status, we will schedule to meet patients at KUMed and sampling will be conducted in private research staff offices.

The study will include no paper data files. The KUMC Department of Preventive Medicine has a well-developed structure for data management. Working data is maintained on a single large file server. Inactive files are moved to archival storage under control of an automated system, itself controlled by a DBMS (Ingres) based request system that ensures that all data movement is appropriately logged and commented. The archival storage is hosted on the institutional mainframe computer, which also supports billing and registration. The use of the mainframe ensures several high-level support functions for the archive system (e.g., storage in separate fire zones, regular copying of data to new media, and guaranteed availability).

The data management will be governed by standard procedures for data security and access. All analyses are logged with respect to IRB authorization, accounting information, principal and co-investigators, statistician, and data analyst involved in the analysis. In order to create a unified data management strategy, we will identify all subjects with a sequentially assigned subject number, and subject initials. To ensure subject confidentiality, no names, social security numbers, hospital or clinic numbers will be included in the shared databases. Names, addresses, telephone numbers, and any other information needed for recruitment, study involvement, and tracking will be obtained and maintained locally by the project personnel. All computer files and systems will be password protected and accessible only by authorized personnel.

Potential Benefits of the Proposed Research to the Participants and Others

Quitting smoking is one of the best things a smoker can do for his or her health regardless of whether or not they participate in this study. The participants will have the opportunity to benefit by making behavioral changes in their smoking or by stopping smoking. Participants who stop smoking will experience invaluable health benefits, and family members of participants who stop smoking would be expected to benefit from reduced/non exposure to second-hand smoke. Participants will be compensated for their time. Following consent and participation in Phase 1 and 2, month 1 follow up survey, staff will mail participants a card pre-loaded with $50. Participants who completed Phase 1 but do not consent AND participants who completed Phase 1 and consent to Phase 1 only will be eligible to receive reimbursement if they provide the required information (name, social security number, etc.) to receive a pre-loaded debit card in the mail from UKanQuit staff. Participants will be reimbursed $25 for each follow-up survey completed and $150 for each salivary sample returned or Carbon Monoxide measurement provided, where applicable, for a maximum total of $375 per participant. Participants will be informed that disbursement of the incentives is not contingent on their smoking status.

Importance of the Knowledge to be Gained

Despite over two decades of intensive tobacco control efforts, one in five Americans continue to smoke. Thus, to address the tobacco use epidemic and its consequent health impact, there is an urgent need to test novel interventions for tobacco treatment. We propose to test the impact of providing all smokers with tobacco

treatment unless they refuse it (OPT OUT) versus current practice—screening for readiness and only offering treatment to smokers who say they are ready to quit (OPT IN). The knowledge to be gained from this research could potentially change the treatment approach offered to hospitalized smokers. The risks involved in gaining this knowledge are reasonable given the potential impact of the knowledge to be gained on smoking cessation treatment.

1. Henningfield JE, Slade J. Tobacco-dependence medications: public health and regulatory issues. *Food and drug law journal*. 1998;53 suppl:75-114.
2. Pierce JP, Gilpin E. How long will today's new adolescent smoker be addicted to cigarettes? *Am J Public Health*. Feb 1996;86(2):253-256.
3. Fiore MC, Jaen CR, Baker TB, et.al. *Treating Tobacco Use and Dependence: 2008 Update. Clinical Practice Guideline*. . Rockville, MD: Department of Health and Human Services. Public Health Service; 2008.
4. Miller WR, Rollnick S. *Motivational Interviewing: Preparing people for change*. 2nd ed. ed. New York: Guilford Press; 2002.
5. Mantler T, Irwin JD, Morrow D. Motivational interviewing and smoking behaviors: a critical appraisal and literature review of selected cessation initiatives. *Psychological reports*. Apr 2012;110(2):445-460.
6. Gonzales D, Rennard SI, Nides M, et al. Varenicline, an alpha4beta2 nicotinic acetylcholine receptor partial agonist, vs sustained-release bupropion and placebo for smoking cessation: a randomized controlled trial. *Jama*. Jul 5 2006;296(1):47-55.
7. Chobanian AV, Bakris GL, Black HR, et al. The Seventh Report of the Joint National Committee on Prevention, Detection, Evaluation, and Treatment of High Blood Pressure: the JNC 7 report. *JAMA*. May 21 2003;289(19):2560-2572.
8. Jamal A, Dube SR, Malarcher AM, et al. Tobacco use screening and counseling during physician office visits among adults--National Ambulatory Medical Care Survey and National Health Interview Survey, United States, 2005-2009. *MMWR Morb Mortal Wkly Rep*. Jun 15 2012;61 Suppl:38-45.
9. Prochaska JO, DiClemente CC. Stages and processes of self-change of smoking: toward an integrative model of change. *J Consult Clin Psychol*. Jun 1983;51(3):390-395.
10. Browning KK, Ferketich AK, Salsberry PJ, Wewers ME. Socioeconomic disparity in provider-delivered assistance to quit smoking. *Nicotine Tob Res*. Jan 2008;10(1):55-61.
11. Freund M, Campbell E, Paul C, et al. Smoking care provision in hospitals: a review of prevalence. *Nicotine Tob Res*. May 2008;10(5):757-774.
12. Centers for Disease C, Prevention. Quitting smoking among adults--United States, 2001-2010. *MMWR Morb Mortal Wkly Rep*. Nov 11 2011;60(44):1513-1519.
13. Richter KP, Ellerbeck EF. It's time to change the default for tobacco treatment. *Addiction*. Oct 16 2014.
14. Thaler RH, Sunstein CR. *Nudge: Improving Decisions About Health, Wealth, and Happiness*. New Haven: Yale University Press; 2008.
15. Qaseem A, Humphrey LL, Sweet DE, Starkey M, Shekelle P, Clinical Guidelines Committee of the American College of P. Oral pharmacologic treatment of type 2 diabetes mellitus: a clinical practice guideline from the American College of Physicians. *Ann Intern Med*. Feb 7 2012;156(3):218-231.
16. National Asthma E, Prevention P. Expert Panel Report 3 (EPR-3): Guidelines for the Diagnosis and Management of Asthma-Summary Report 2007. *The Journal of allergy and clinical immunology*. Nov 2007;120(5 Suppl):S94-138.
17. Johnson EJ, Steffel M, Goldstein DG. Making better decisions: from measuring to constructing preferences. *Health Psychol*. Jul 2005;24(4 Suppl):S17-22.
18. Samuelson W, Zeckhauser R. Status quo bias in decisionmaking. *journal of Risk and Uncertainty*. 1988;1:7-59.
19. Bellman S, Johnson EJ, Lohse GL. To opt-in or opt-out? It depends on the question. *Communications of the ACM*. 2001;44(2):25-27.

20. Johnson EJ, Goldstein D. Medicine. Do defaults save lives? *Science*. Nov 21 2003;302(5649):1338-1339.
21. Olds RS. Informed-consent issues with adolescent health behavior research. *American journal of health behavior*. Nov-Dec 2003;27 Suppl 3:S248-263.
22. Corporation WD. Corporate Responsibility Report. 2008; http://disney.go.com/crreport/childrenandfamily/positivedevelopment/kidshealthandnutrition.html.
23. McKenzie CR, Liersch MJ, Finkelstein SR. Recommendations implicit in policy defaults. *Psychological science*. May 2006;17(5):414-420.
24. Van De Veer D. *Paternilistic Intervention: The Moral Bounds on Benevolence*. Princeton: Princeton University Press; 1986.
25. Centers for Disease C, Prevention. Vital signs: current cigarette smoking among adults aged >or=18 years --- United States, 2009. *MMWR Morb Mortal Wkly Rep*. Sep 10 2010;59(35):1135-1140.
26. Ockene JK. Physician-delivered interventions for smoking cessation: strategies for increasing effectiveness. *Prev Med*. Sep 1987;16(5):723-737.
27. Stitzer ML. Combined behavioral and pharmacological treatments for smoking cessation. *Nicotine Tob Res*. 1999;1 Suppl 2:S181-187; discussion S207-110.
28. Rigotti NA, Clair C, Munafo MR, Stead LF. Interventions for smoking cessation in hospitalised patients. *Cochrane Database Syst Rev*. 2012;5:CD001837.
29. Stead LF, Perera R, Lancaster T. Telephone counselling for smoking cessation. *Cochrane Database Syst Rev*. 2006;3:CD002850.
30. Borland R, Segan CJ. The potential of quitlines to increase smoking cessation. *Drug Alcohol Rev*. Jan 2006;25(1):73-78.
31. Bailey L. An Overview of NAQC. National Network of Tobacco Cessation Quitlines -- 2006 Regional Meeting -- Western Region; May 1-2, 2006, 2006; San Diego, CA.
32. Ossip-Klein DJ, McIntosh S. Quitlines in North America: evidence base and applications. *Am J Med Sci*. Oct 2003;326(4):201-205.
33. Swartz SH, Cowan TM, Klayman JE, Welton MT, Leonard BA. Use and effectiveness of tobacco telephone counseling and nicotine therapy in Maine. *Am J Prev Med*. Nov 2005;29(4):288-294.
34. Pisinger C, Vestbo J, Borch-Johnsen K, Jorgensen T. It is possible to help smokers in early motivational stages to quit. The Inter99 study. *Prev Med*. Mar 2005;40(3):278-284.
35. Ellerbeck EF, Mahnken JD, Cupertino AP, et al. Effect of varying levels of disease management on smoking cessation: a randomized trial. *Ann Intern Med*. Apr 7 2009;150(7):437-446.
36. Hughes JR, Keely JP, Fagerstrom KO, Callas PW. Intentions to quit smoking change over short periods of time. *Addict Behav*. May 2005;30(4):653-662.
37. Ferguson SG, Shiffman S, Gitchell JG, Sembower MA, West R. Unplanned quit attempts--results from a U.S. sample of smokers and ex-smokers. *Nicotine Tob Res*. Jul 2009;11(7):827-832.
38. Pisinger C, Vestbo J, Borch-Johnsen K, Thomsen T, Jorgensen T. Acceptance of the smoking cessation intervention in a large population-based study: the Inter99 study. *Scandinavian journal of public health*. 2005;33(2):138-145.
39. Aveyard P, Begh R, Parsons A, West R. Brief opportunistic smoking cessation interventions: a systematic review and meta-analysis to compare advice to quit and offer of assistance. *Addiction*. Jun 2012;107(6):1066-1073.
40. Slama K, Redman S, Perkins J, Reid AL, Sanson-Fisher RW. The effectiveness of two smoking cessation programmes for use in general practice: a randomised clinical trial. *BMJ*. Jun 30 1990;300(6741):1707-1709.
41. Hodgins DC. Weighing the pros and cons of changing change models: a comment on West (2005). *Addiction*. Aug 2005;100(8):1042-1043; author reply 1048-1050.

42. Ryan RM. Psychological needs and the facilitation of integrative processes. *Journal of personality*. Sep 1995;63(3):397-427.

43. Halpern SD, Ubel PA, Asch DA. Harnessing the power of default options to improve health care. *N Engl J Med*. Sep 27 2007;357(13):1340-1344.

44. Campbell DT, Stanley JC. *Experimental and Quasi-Experimental Designs for Research*. Hopewell, NJ: Houghton Mifflin Company; 1963.

45. Berry DA. Bayesian clinical trials. *Nature reviews. Drug discovery*. Jan 2006;5(1):27-36.

46. Connor JT, Elm JJ, Broglio KR, Esett, Investigators A-I. Bayesian adaptive trials offer advantages in comparative effectiveness trials: an example in status epilepticus. *Journal of clinical epidemiology*. Aug 2013;66(8 Suppl):S130-137.

47. Faseru B, Turner M, Casey G, et al. Evaluation of a hospital-based tobacco treatment service: outcomes and lessons learned. *Journal of hospital medicine : an official publication of the Society of Hospital Medicine*. Apr 2011;6(4):211-218.

48. Faseru B, Yeh HW, Ellerbeck EE, Befort C, Richter KP. Prevalence and predictors of tobacco treatment in an academic medical center. *Joint Commission journal on quality and patient safety / Joint Commission Resources*. Nov 2009;35(11):551-557.

49. Richter KP, Faseru B, Mussulman LM, et al. Using "warm handoffs" to link hospitalized smokers with tobacco treatment after discharge: study protocol of a randomized controlled trial. *Trials*. 2012;13:127.

50. Stoltzfus K, Hunt S, Ayars C, Carlini B, Rabius V, Richter K. A pilot trial of proactive versus reactive referral to tobacco quitlines. *Journal of Smoking Cessation*. 2011;6(4):211-218.

51. Catley D, Harris KJ, Goggin K, et al. Motivational Interviewing for encouraging quit attempts among unmotivated smokers: study protocol of a randomized, controlled, efficacy trial. *BMC public health*. 2012;12:456.

52. Baker TB, Mermelstein R, Collins LM, et al. New methods for tobacco dependence treatment research. *Annals of behavioral medicine : a publication of the Society of Behavioral Medicine*. Apr 2011;41(2):192-207.

53. Hughes JR, Keely J, Naud S. Shape of the relapse curve and long-term abstinence among untreated smokers. *Addiction*. Jan 2004;99(1):29-38.

54. Garvey AJ, Bliss RE, Hitchcock JL, Heinold JW, Rosner B. Predictors of smoking relapse among self-quitters: a report from the Normative Aging Study. *Addict Behav*. 1992;17(4):367-377.

55. Commission TJ. Performance Measurement Initiatives: Screening and Treating Tobacco and Alcohol Use. 2009; http://www.jointcommission.org/PerformanceMeasurement/PerformanceMeasurement/Screening+and+Treating+Tobacco+and+Alcohol+Use.htm. Accessed January 5, 2010.

56. Services CfMM. Readmissions Reduction Program. 2013; http://cms.gov/Medicare/Medicare-Fee-for-Service-Payment/AcuteInpatientPPS/Readmissions-Reduction-Program.html. Accessed December 26, 2013.

57. Association AL. Tobacco Cessation Treatment is an Essential Health Benefit. 2012; http://www.lung.org/stop-smoking/tobacco-control-advocacy/reports-resources/2012/factsheet-essential-health-benefits.pdf. Accessed January 15, 2014.

58. Mussulman L, Ellerbeck EF, Cupertino AP, et al. Design and participant characteristics of a randomized-controlled trial of telemedicine for smoking cessation among rural smokers. *Contemporary clinical trials*. Apr 24 2014;38(2):173-181.

59. Hughes JR, Keely JP, Niaura RS, Ossip-Klein DJ, Richmond RL, Swan GE. Measures of abstinence in clinical trials: issues and recommendations. *Nicotine Tob Res*. Feb 2003;5(1):13-25.

60. Biochemical verification of tobacco use and cessation. *Nicotine Tob Res*. May 2002;4(2):149-159.

61. Benowitz NL, Bernert JT, Caraballo RS, Holiday DB, Wang J. Optimal serum cotinine levels for distinguishing cigarette smokers and nonsmokers within different racial/ethnic groups in the United States between 1999 and 2004. *Am J Epidemiol*. Jan 15 2009;169(2):236-248.
62. Hyland A, Cummings KM, Lynn WR, Corle D, Giffen CA. Effect of proxy-reported smoking status on population estimates of smoking prevalence. *Am J Epidemiol*. Apr 15 1997;145(8):746-751.
63. Gilpin EA, Pierce JP, Cavin SW, et al. Estimates of population smoking prevalence: self-vs proxy reports of smoking status. *Am J Public Health*. Oct 1994;84(10):1576-1579.
64. Chen Y, Rennie DC, Dosman JA. The reliability of cigarette consumption reports by spousal proxies. *Am J Public Health*. Dec 1995;85(12):1711-1712.
65. Williams GC, McGregor H, Sharp D, et al. A Self-Determination Multiple Risk Intervention Trial to Improve Smokers' Health. *J Gen Intern Med*. Sep 25 2006.
66. Foulds J, Steinberg MB, Williams JM, Ziedonis DM. Developments in pharmacotherapy for tobacco dependence: past, present and future. *Drug Alcohol Rev*. Jan 2006;25(1):59-71.
67. Knoll MA. The role of behavioral economics and behavioral decision making in Americans' retirement savings decisions. *Social security bulletin*. 2010;70(4):1-23.
68. Baron RM, Kenny DA. The Moderator-Mediator Variable Distinction in Social Psychological Research: Conceptual, Stategic, and Statistical Considerations. *Journal of Peronality and Social Psychology*. 1986;51(6):1173-1182.
69. Berry SM, Carlin BP, Lee JJ, Muller P. *Bayesian Adaptive Methods for Clinical Trials*. New York: CRC Press; 2011.
70. JT C, JJ E, KR B. Bayesian Adaptive Trials for Comparative Effectiveness Research: An Example in Status Epilepticus. *Journal of clinical epidemiology*. In Press.
71. Gold MR, Siegel JE, Russell LB, Weinstein MC. *Cost-Effectiveness in Health and Medicine*. New York: Oxford University Press; 1996.
72. Gold MR, Siegel JE, Russell LB, Weinstein MC, eds. *Cost-Effectiveness in Health and Medicine*. New York: Oxford University Press; 1996.
73. Haddix AC, Teutsch SM, Corso PS, eds. *Prevention Effectiveness: A Guide to Decision Analysis and Economic Evaluation*. 2nd Edition ed. Oxford: Oxford University Press; 2003.
74. Zelen M. A new design for randomized clinical trials. *New England Journal of Medicine*. 1979;300:1242-1245.
75. *Joint Commission on Accreditation of Healthcare Organizations (JCAHO). (2004). Performance Measurement Initiatives. Retrieved March 30, 2009, from* http://www.jointcommission.org/PerformanceMeasurement/PerformanceMeasurement.
76. Faden RR, Beauchamp TL, Kass NE. Informed consent, comparative effectiveness, and learning health care. *N Engl J Med*. Feb 20 2014;370(8):766-768.
