# Supplementary material for: Desire to Quit Smoking, Opt-Out Tobacco Treatment, and Cessation: A Secondary Analysis of a Randomized Clinical Trial
Source: JAMA Netw Open. 2024 Sep 17;7(9):e2433802. doi: 10.1001/jamanetworkopen.2024.33802 (PMC11409148; doi:10.1001/jamanetworkopen.2024.33802)
Supplement: Supplement 2. — Data Sharing Statement [file jamanetwopen-e2433802-s002.pdf]

Data Sharing Statement

Gajewski. Desire to Quit Smoking, Opt-Out Tobacco Treatment, and Cessation: A Secondary Analysis of a Randomized Clinical Trial. *JAMA Netw Open*. Published online September 17, 2024. doi:10.1001/jamanetworkopen.2024.33802

Data
Data available: No

Additional Information
Explanation for why data not available: The data sharing statement for this study is in the JAMA Internal Medicine paper referenced
